# A20 and the noncanonical NF-**κ**B pathway are key regulators of neutrophil recruitment during fetal ontogeny

**DOI:** 10.1172/jci.insight.155968

**Published:** 2023-02-22

**Authors:** Ina Rohwedder, Lou Martha Wackerbarth, Kristina Heinig, Annamaria Ballweg, Johannes Altstätter, Myriam Ripphahn, Claudia Nussbaum, Melanie Salvermoser, Susanne Bierschenk, Tobias Straub, Matthias Gunzer, Marc Schmidt-Supprian, Thomas Kolben, Christian Schulz, Averil Ma, Barbara Walzog, Matthias Heinig, Markus Sperandio

**Affiliations:** 1 Institute of Cardiovascular Physiology and Pathophysiology, Walter-Brendel-Center of Experimental Medicine, Biomedical Center Munich, LMU Munich, Planegg-Martinsried, Germany.; 2 Division of Neonatology, Department of Pediatrics, Dr. von Hauner Children’s Hospital, LMU Munich, Munich, Germany.; 3 Core Facility Bioinformatics, Biomedical Center Munich, Faculty of Medicine, LMU Munich, Planegg-Martinsried, Germany.; 4 Institute for Experimental Immunology and Imaging, University of Duisburg-Essen, Essen, Germany.; 5 Leibniz-Institut für Analytische Wissenschaften - ISAS - e.V., Dortmund, Germany.; 6 Department of Hematology and Medical Oncology, TU Munich, Munich, Germany.; 7 Department of Gynecology and Obstetrics and; 8 Medical Clinic I, University Hospital, LMU Munich, Munich, Germany.; 9 Department of Medicine, University of California, San Francisco, San Francisco, California, USA.; 10 Institute of Computational Biology, Helmholtz Munich, Munich, Germany.; 11 Department of Informatics, TU Munich, Munich, Germany.

**Keywords:** Inflammation, Innate immunity, NF-kappaB, Neutrophils

## Abstract

Newborns are at high risk of developing neonatal sepsis, particularly if born prematurely. This has been linked to divergent requirements the immune system has to fulfill during intrauterine compared with extrauterine life. By transcriptomic analysis of fetal and adult neutrophils, we shed new light on the molecular mechanisms of neutrophil maturation and functional adaption during fetal ontogeny. We identified an accumulation of differentially regulated genes within the noncanonical NF-κB signaling pathway accompanied by constitutive nuclear localization of RelB and increased surface expression of TNF receptor type II in fetal neutrophils, as well as elevated levels of lymphotoxin α in fetal serum. Furthermore, we found strong upregulation of the negative inflammatory regulator A20 (*Tnfaip3*) in fetal neutrophils, which was accompanied by pronounced downregulation of the canonical NF-κB pathway. Functionally, overexpressing A20 in Hoxb8 cells led to reduced adhesion of these neutrophil-like cells in a flow chamber system. Conversely, mice with a neutrophil-specific A20 deletion displayed increased inflammation in vivo. Taken together, we have uncovered constitutive activation of the noncanonical NF-κB pathway with concomitant upregulation of A20 in fetal neutrophils. This offers perfect adaption of neutrophil function during intrauterine fetal life but also restricts appropriate immune responses particularly in prematurely born infants.

## Graphical abstract

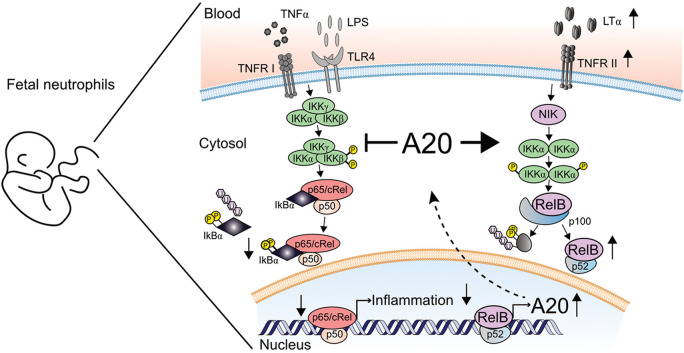

## Introduction

Prematurely born infants are highly susceptible to bacterial, fungal, and viral infections and have increased morbidity and mortality from neonatal sepsis compared with term babies (1, 2). This clinical observation reflects the contrasting requirements the immune system has to fulfill to guarantee normal growth and development during intrauterine life on one hand and, on the other hand, to cope with the challenges and threats infants are exposed to in the outside world after birth (3, 4). For the innate immune system, key players in host defense are neutrophils, which, upon an inflammatory stimulus, are attracted to the site of inflammation by a well-orchestrated recruitment process that finally leads to their extravasation from the bloodstream into inflamed tissue (5, 6). This recruitment process is severely restricted during early stages of fetal development when compared with adults (7, 8). As a functional consequence, murine and human fetal neutrophils display a limited capacity to roll and adhere in vitro and in vivo. Interestingly, exposure to the extrauterine environment as in prematurely born infants does not stimulate neutrophil function, but instead, neutrophil maturation seems to be regulated by an intrinsic molecular program (7). Recently, using various systems biology approaches, several studies have investigated immune system development during the neonatal period (9–11). Besides an increase in type I IFN–related functions, an ontogenetic control of neutrophil signaling was suggested targeting Toll-like receptor– and IL-1–dependent signaling, both of which affect the NF-κB signaling pathways (9).

The NF-κB family of transcription factors is ubiquitously expressed and regulates numerous targets particularly in immune cells (12). Two major signaling pathways exist: the canonical and the noncanonical. The canonical signaling pathway is activated downstream of inflammatory surface receptors on immune cells including Toll-like receptors (TLRs) or the tumor necrosis factor–α (TNF-α) receptor and leads to nuclear translocation of the NF-κB subunit p65. It initiates the rapid but transient activation of gene transcription of proinflammatory cytokines and therefore induces and prolongs the inflammatory response. In contrast, RelB, known as the primary effector of the noncanonical NF-κB signaling pathway, initiated by a diverse range of ligands and receptors, like lymphotoxin α receptor/lymphotoxin α1β2 (LTβR/LT-α1β2) or TNF receptor type II/LT-α (TNFRII/LT-α), elicits a slow and persistent activation of gene transcription (13). While the noncanonical NF-κB signaling pathway is well known for its importance in lymph node development during fetal ontogeny (14), it also functions as a fine-tuning mechanism for inflammatory responses. This was shown by chromatin immunoprecipitation (ChIP) sequencing that revealed numerous RelB downstream targets, among them important immune modulators such as interleukin 1 receptor associated kinase 3 (IRAK-3) and the ubiquitin modifying enzyme A20 (gene name: tumor necrosis factor-alpha-induced protein 3, *TNFAIP3*) (15). A20 is a key negative feedback inhibitor of the canonical NF-κB signaling pathway exerting its immunomodulatory function through its ubiquitin modifying properties (16–18). Accordingly, A20 deficiency in mice results in multiorgan inflammation and premature death (19). Further studies using conditional knockout mice focused on the role of A20 in innate immune cells. Mice with an A20 deficiency (*Tnfaip3^–/–^* mice) in the myeloid compartment develop spontaneous polyarthritis with sustained proinflammatory cytokine production (20).

Using a whole-transcriptome analysis approach, we aimed to investigate the molecular mechanisms regulating neutrophil maturation during fetal development and found a robust upregulation of RelB target genes in fetal neutrophils, including A20. Furthermore, we could observe nuclear accumulation of RelB in fetal neutrophils, suggesting constitutively active noncanonical NF-κB signaling and A20 as a key modifier of neutrophil function during fetal ontogeny.

## Results

### Differential gene expression signatures in human fetal and adult neutrophils.

**Figure 1:**
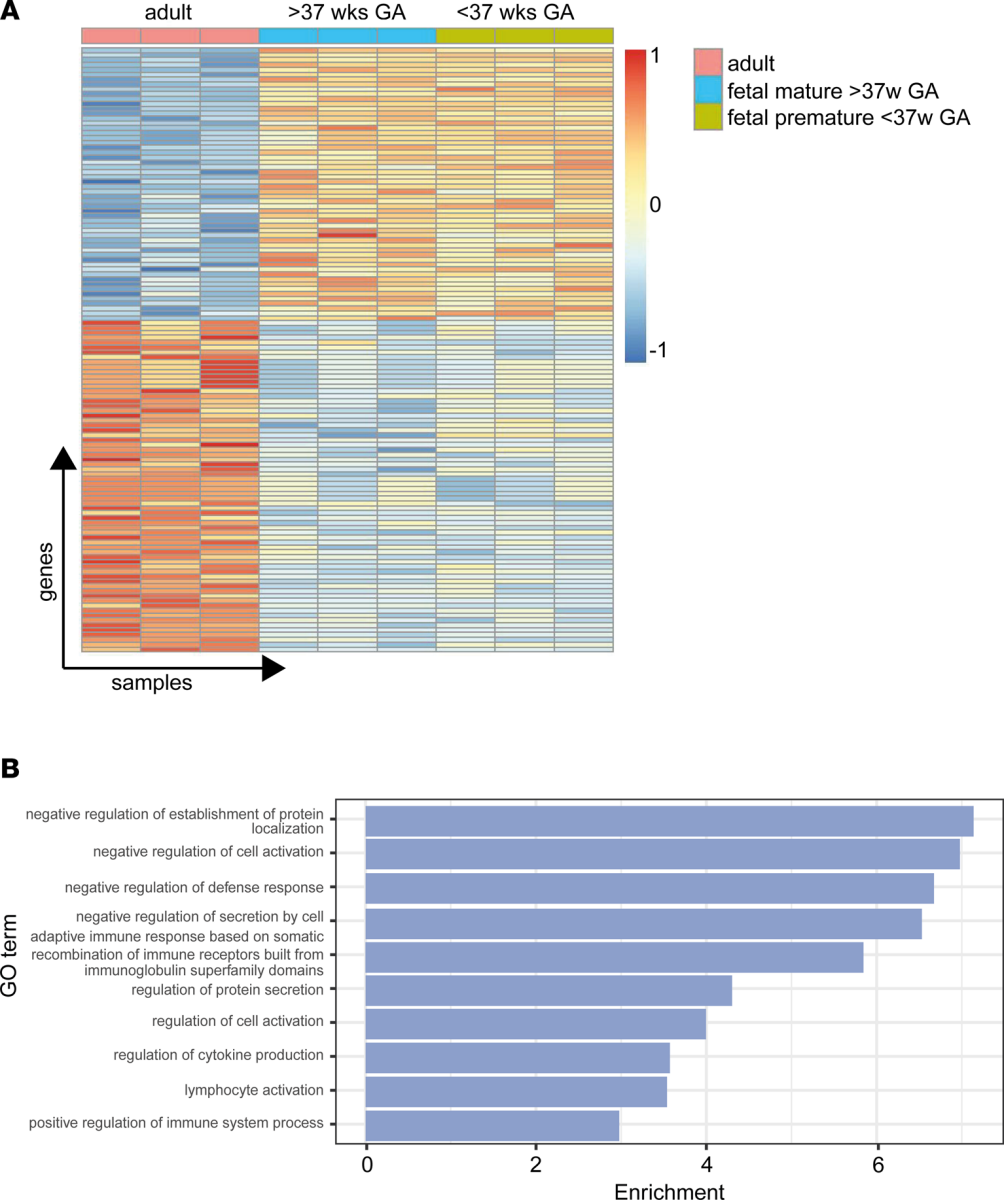
Differential gene expression signatures in human fetal and adult neutrophils. (**A**) Neutrophils were isolated from peripheral blood of adult healthy donors and umbilical cord blood samples from mature fetuses (gestational age > 37 weeks) or premature fetuses (gestational age < 37 weeks) (*n* = 3 per group). Differentially regulated genes between the samples are shown, each column representing 1 sample and each line 1 gene. Upregulated genes are depicted in red, whereas downregulated genes are depicted in blue. (**B**) Differentially expressed genes were used for gene set enrichment analysis. The bar plot shows the fold enrichment (*x* axis) of the fraction of members of the gene set within all differentially expressed genes divided by the fraction of members of the gene set in the genome-wide background set for each of the gene sets (*y* axis), which are among the top 10 of all gene sets with FDR < 5%. GO, Gene Ontology.

Fetal neutrophils from early embryonic stages display a reduced capacity to react to inflammatory stimuli, thus leaving the organism highly vulnerable to invading pathogens or sterile inflammation. This study aimed to investigate the underlying molecular mechanisms regulating fetal neutrophil function during ontogeny. We therefore performed whole-transcriptome analysis of human fetal cord blood–derived neutrophils from premature (<37 weeks of gestation) and mature (>37 weeks of gestation) infants and compared their gene expression profiles with neutrophils from healthy adult donors (Figure 1A). Flow cytometric analysis and cytospin were used to ensure that comparable cell populations of human adult and mature fetal neutrophils were included in the analysis (Supplemental Figure 1, A and B; supplemental material available online with this article; https://doi.org/10.1172/jci.insight.155968DS1). We identified 124 differentially regulated genes (FDR < 5%, Figure 1A); 56 were upregulated and 68 were downregulated in fetal versus adult neutrophils. To understand the biological significance underlying these differentially regulated genes and provide a rough overview, we applied WEB-based Gene SeT AnaLysis Toolkit (WebGestalt), which allowed us to group our candidate genes according to their function and association with biological processes (Figure 1B and Supplemental Table 1). Strikingly, the analysis revealed that most prominent changes in identified gene sets occurred for those sets of genes with negative regulatory functions in cellular defense and activation as well as protein trafficking.

### The noncanonical NF-κB subunit RelB determines a specific gene expression signature in fetal human neutrophils.

**Figure 2:**
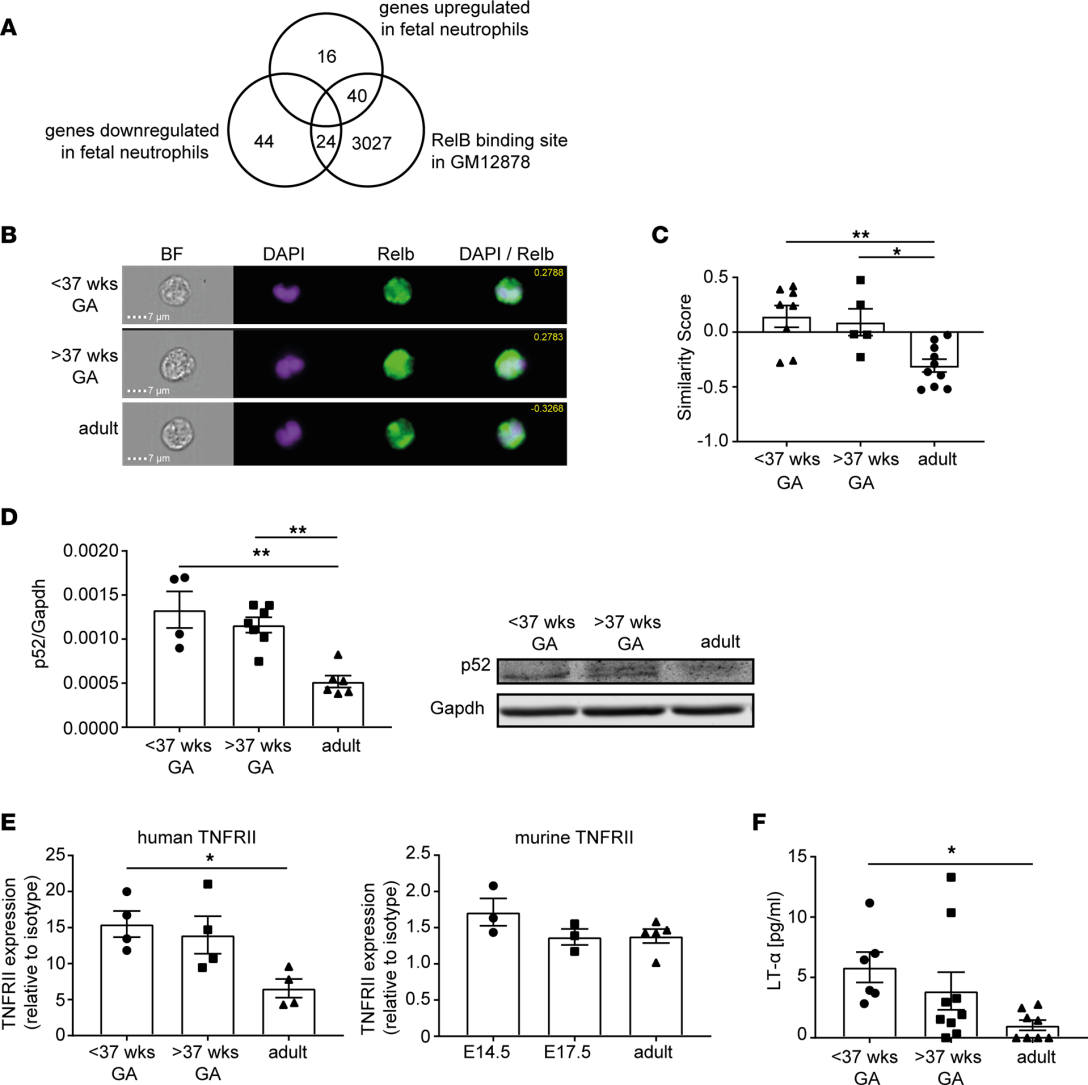
The noncanonical NF-κB subunit RelB determines a specific gene expression signature in fetal human neutrophils. (**A**) Venn diagram representing RelB-regulated genes within all genes up- and downregulated in adult compared with fetal neutrophils. Data were correlated with GM12878 human lymphoblastoid B cells (published data set) (15). (**B**) Imaging flow cytometry was performed on cord blood neutrophils from premature (gestational age < 37 weeks) and mature (gestational age > 37 weeks) fetal samples and peripheral blood from adult healthy donors. Representative pictures of fetal and adult neutrophils are shown as bright-field image, DAPI, NF-κB subunit RelB, and a DAPI/RelB overlay. Respective similarity scores are displayed. Scale bar: 7 μm. (**C**) Quantification of nuclear RelB. Similarity score defines overlap of nuclear DAPI signal and the respective NF-κB subunit. All data are presented as mean ± SEM. (*P < 0.05, **P < 0.005, *n* = 5–10.) Ordinary 1-way ANOVA with Tukey’s multiple comparisons test. (**D**) Western blot and respective quantitative analysis of p52 in isolated neutrophils from premature (gestational age < 37 weeks), mature (gestational age > 37 weeks), and adult samples. Band intensity was normalized to Gapdh. All data are presented as mean ± SEM (**P < 0.005, *n* = 4–7). Ordinary 1-way ANOVA with Dunnett’s multiple comparisons test. (**E**) Flow cytometry analysis of TNFRII expression on human and murine neutrophils out of whole blood from indicated gestational ages. Median fluorescence intensity normalized to isotype control is displayed. All data are presented as mean ± SEM (*P < 0.05, *n* = 3–5). Ordinary 1-way ANOVA with Dunnett’s multiple comparisons test. (**F**) LT-α protein levels in cord blood serum from premature (gestational age < 37 weeks) and mature infants (gestational age >37 weeks) and from whole blood of adult healthy donors by quantitative ELISA. Values are displayed in pg/mL. All data are presented as mean ± SEM. (*P < 0.05, *n* = 6–9.) Ordinary 1-way ANOVA with Dunnett’s multiple comparisons test.

Next, we analyzed the differentially regulated genes found in our transcriptomic screen in more detail. We could associate several of the differentially regulated genes (including *TNFAIP3*, *IRAK3*, *RELB*) to the NF-κB signaling pathway. This prompted us to systematically assess whether binding sites of NF-κB subunits in GM12878 human lymphoblastoid B cells (15) were overrepresented at promoters of genes that were upregulated or downregulated in fetal neutrophils in comparison with adult neutrophils. RelB was the subunit with strongest overrepresentation in promoters of upregulated genes (*P* = 5.19 × 10^–7^ Fisher’s exact test, OR = 4.0, Supplemental Table 2). Using this approach, we were able to identify 40 RelB target genes that were upregulated in fetal neutrophils compared with their adult counterparts and an additional 24 RelB target genes displaying the opposite signature with lower expression in fetal neutrophils (Figure 2A and Supplemental Table 3). This means that 71% of all fetal upregulated genes and 35% of all fetal downregulated genes are associated with the RelB pathway.

Upon activation of the noncanonical NF-κB signaling pathway, the transcription factor RelB is translocated from the cytoplasm into the nucleus to induce gene transcription. In order to test for baseline activation of the noncanonical pathway, we analyzed RelB localization in fetal and adult human neutrophils using imaging flow cytometry (Amnis). This allows us to assess the subcellular localization of RelB on a large cell population. We were able to detect colocalization of RelB to the DAPI-stained nucleus of premature and mature fetal neutrophils, while this was significantly reduced in adult neutrophils (Figure 2, B and C). In addition, we could see the same increased colocalization of RelB to the DAPI-stained nucleus in murine fetal neutrophils compared to adult cells (Supplemental Figure 1C). Furthermore, the canonical NF-κB subunit p65 was not differentially colocalizing to the nucleus in fetal or adult neutrophils, suggesting an exclusive baseline activation of the noncanonical NF-κB signaling pathway in human fetal neutrophils (Supplemental Figure 1D). We then quantified total RelB protein in our samples and could observe no significant alterations in overall RelB expression in fetal versus adult neutrophils, although a tendency toward increased RelB amount was detected in samples from premature infants (Supplemental Figure 1E). In resting cells, RelB activation and translocation is inhibited by its binding to p100. Upon activation of the noncanonical NF-κB signaling pathway, p100 is in part proteasomally degraded into p52, and subsequently, as a dimer with RelB, translocated into the nucleus. In accordance with our previous results, we detected significantly higher baseline levels of cleaved p52 in fetal neutrophils compared with adult neutrophils by Western blot analysis, with the highest values in early premature samples under 37 weeks of gestational age (Figure 2D). As these results indicate that in fetal neutrophils NF-κB signaling is shifted toward the noncanonical pathway, we were interested in upstream factors inducing this pathway. While TLR4 and the TNF receptor are well-known inducers of the canonical NF-κB signaling pathway, there are also a variety of receptors that differentially induce the noncanonical pathway, including CD40, Ox40, RANK, LTβR, and TNFRII being expressed on neutrophils. Using flow cytometry analysis, we could detect expression of TNFRII (Figure 2E) and LTβR (Supplemental Figure 2A) on fetal and adult human as well as murine neutrophils. Interestingly, TNFRII displayed significantly higher surface expression levels in human samples obtained at a gestational age under 37 weeks compared with adult, with a similar tendency, although not significant, in murine samples (Figure 2E). One of the ligands for TNFRII is LT-α, well known for its role in the development of secondary lymphatic tissue (21). We analyzed serum levels of LT-α in cord blood serum of human premature and mature infants as well as in adult serum using quantitative ELISA. We detected significantly higher LT-α serum levels in cord blood of premature and mature infants than in samples from adults (Figure 2F), suggesting that the LT-α/TNFRII axis might play a critical role in the observed elevated baseline activation of the immunomodulatory noncanonical NF-κB signaling pathway in fetal neutrophils.

### Downregulation of the canonical NF-κB signaling pathway in fetal neutrophils.

**Figure 3:**
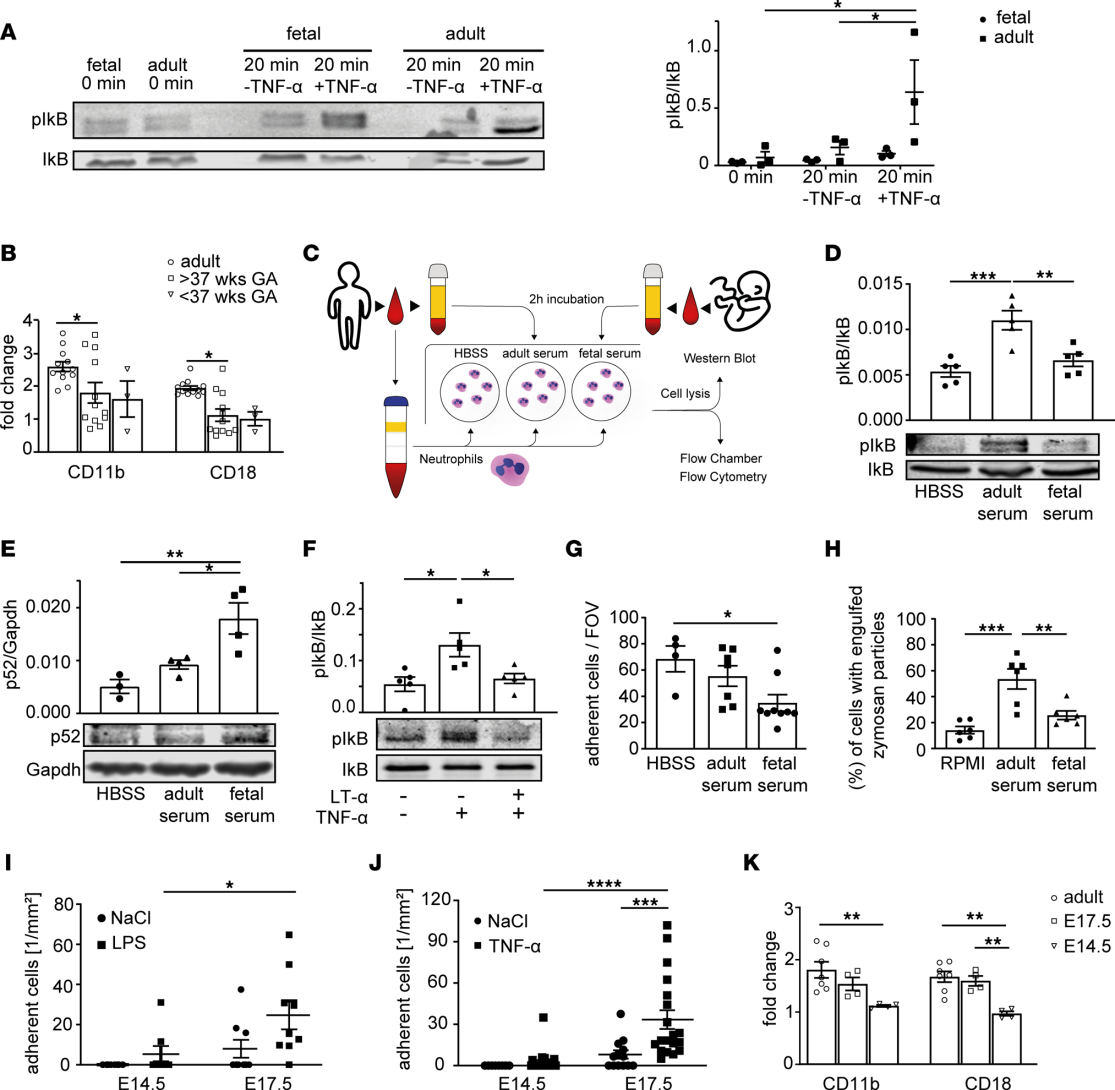
Downregulation of the canonical NF-κB signaling pathway in fetal neutrophils. (**A**) Western blot of IκB phosphorylation after TNF-α stimulation in fetal (gestational age > 37 weeks) and adult human neutrophils. Normalization to IκB. (*P < 0.05, *n* = 3.) Two-way ANOVA with Tukey’s multiple comparisons. (**B**) Quantitative analysis of Mac-1 levels after 2-hour TNF-α stimulation in human adult, mature, and premature fetal neutrophils by flow cytometry. Fold-change: normalization to control. (*P < 0.05, *n* = 3–12.) Two-way ANOVA with Tukey’s multiple comparisons. (**C**) Workflow: adult blood neutrophils after 2-hour stimulation with HBSS or RPMI, fetal or adult serum. (**D**) Western blot analysis of phospho-IκB in adult neutrophils after stimulation with adult or fetal serum or HBSS. Normalization to IκB. (**P < 0.005, ***P < 0.001; *n* = 3–5.) Ordinary 1-way ANOVA with Tukey’s multiple comparisons. (**E**) Western blot analysis of p52 in adult neutrophils after stimulation with adult or fetal serum. Normalization to Gapdh. (*P < 0.05, **P < 0.005, *n* = 3–4.) Ordinary 1-way ANOVA with Tukey’s multiple comparisons. (**F**) Western blot analysis of IκB phosphorylation after incubation of human adult neutrophils with LT-α, followed by TNF-α stimulation. Normalized to IκB. (*P < 0.05; *n* = 5.) Ordinary 1-way ANOVA with Tukey’s multiple comparisons. (**G**) Adherent cells per FOV in flow chambers coated with rhE selectin, rhICAM-1, and rhIL-8 after stimulation of human adult neutrophils with adult or fetal serum. (*P < 0.05; *n* = 4-9.) Ordinary 1-way ANOVA with Tukey’s multiple comparisons. (**H**) Quantitative analysis of phagocytosis by human neutrophils measuring engulfed Zymosan particles by flow cytometry after stimulation with adult or fetal serum. (**P < 0.005, ***P < 0.001; *n* = 6.) Ordinary 1-way ANOVA with Tukey’s multiple comparisons. (**I** and **J**) Quantified adherent cells in yolk sac vessels after LPS (**I**) or TNF-α (**J**) stimulation, compared with control. (*P < 0.05, ***P < 0.001, ****P < 0.0001, *n* = 3–8 mice.) Two-way ANOVA with Šídák’s (**I**) or Tukey’s (**J**) multiple comparisons. (**K**) Quantitative analysis of Mac-1 surface levels after 2-hour TNF-α stimulation in murine adult and E14.5 and E17.5 fetal neutrophils by flow cytometry. Fold-change: normalization to control. (**P < 0.005; *n* = 3–12.) Two-way ANOVA with Tukey’s multiple comparisons. All data are presented as mean ± SEM (**A**, **B**, and **D**–**K**).

Because of the described immune-modulatory function of the noncanonical NF-κB signaling pathway and its antagonizing effects on canonical NF-κB signaling, we wanted to test whether fetal neutrophils also display reduced inflammation-driven activation of the canonical NF-κB pathway. To address this, we stimulated fetal and adult human neutrophils for 20 minutes with 10 ng/mL TNF-α, which induces canonical signaling via the TNF receptor. Upon activation of the TNF receptor, a series of kinases is activated, resulting in the phosphorylation of IκBα, IκBβ, and IκBγ, leading to their proteasomal degradation and release of p65 and p50 heterodimers. While TNF-α stimulation resulted in a robust phosphorylation of IκBα in adult neutrophils (Figure 3A), we were unable to observe a similar increase in phosphorylation in fetal samples, indicating that fetal neutrophils are less capable of activating the canonical signaling cascade upon stimulation. This observation was not due to a differential expression of the TNF1 receptor (TNFRI) on the neutrophil surface (Supplemental Figure 2B), suggesting an intracellular regulatory mechanism. Additionally, stimulation with TNF-α was unable to induce upregulation of Mac-1 (α_M_β_2_, CD11b/CD18) surface levels in human fetal neutrophils to the same extent as in adult neutrophils (Figure 3B). Surface expressed Mac-1 has been demonstrated to be critical for neutrophil recruitment into inflamed tissue (22). Hypothesizing that factors in fetal serum like LT-α keep fetal neutrophils in a less activatable state, we stimulated adult neutrophils with fetal or adult serum and analyzed the levels of phosphorylated IκBα and p52 as well as neutrophil effector functions (Figure 3C). Incubating neutrophils with fetal serum showed diminished phosphorylation of IκBα compared with stimulation with adult serum. When being stimulated with fetal serum, p52 protein levels increased compared with controls (Figure 3, D and E), indicating that fetal serum induces a shift in adult neutrophils toward the noncanonical signaling pathway, keeping neutrophils in a rather unresponsive state toward inflammatory stimuli. We performed the same experiment as described before on fetal human neutrophils and could see that adult human serum was able to switch fetal neutrophils to a proinflammatory state with upregulated phosphorylated IκB levels (Supplemental Figure 2C). This again reinforces our hypothesis that factors like LT-α in the fetal serum are able to dampen the inflammatory NF-κB–induced response. Interestingly, incubation of human adult neutrophils with recombinant human LT-α for 2 hours was also able to prevent canonical NF-κB signaling via TNF-α (Figure 3F), as shown by strongly reduced phosphorylation levels of IκBα.

Next, we investigated if incubation of human adult neutrophils with fetal serum is also affecting other neutrophil functions and tested neutrophil adhesion using flow chambers coated with recombinant human intercellular adhesion molecule 1 (rhICAM-1)/rhE selectin and rhCXCL8 as a substrate for adhesion. These experiments showed that adult neutrophils incubated with fetal serum displayed reduced adhesion in the flow chamber compared with cells incubated with adult serum, indicating that fetal serum has the capacity to reduce neutrophil adhesion under flow (Figure 3G).

Using the Zymosan uptake assay, we then analyzed phagocytosis of human adult neutrophils after incubation with fetal serum. Again, fetal serum incubation was able to reduce phagocytosis of Zymosan by adult neutrophils (Figure 3H), providing evidence that various neutrophil functions are altered in the presence of fetal serum. Blocking TNFRII on human adult neutrophils using an anti–human TNFRII antibody was able to prevent the inhibitory activity of fetal serum, since phagocytic activity of adult neutrophils stimulated with fetal serum was significantly increased after blocking of TNFRII beforehand. Adult neutrophils in the presence of adult serum after blocking the TNFRII receptor did not show increased phagocytic activity, verifying an important role of the LT-α/TNFRII axis in modulating fetal neutrophil function (Supplemental Figure 2D). Next, we applied an in vivo model of fetal inflammation and performed intravital imaging experiments on neutrophil adhesion in inflamed mouse yolk sac vessels 2 hours after intrauterine LPS (100 μg) stimulation. These experiments were conducted with *Lyz2^GFP^* mice (23), where neutrophils harbor a bright GFP signal. LPS via binding to TLR4 is one of the main activators of the canonical NF-κB signaling pathway, inducing a proinflammatory MyD88-dependent transcriptional program that consequently results in the activation of neutrophils and endothelial cells, inducing neutrophil adhesion to the inflamed endothelium. While in E14.5 yolk sac vessels only very few leukocytes interacted with the vessel wall after LPS stimulation (Supplemental Video 1 and Figure 3I), we were able to detect significantly higher numbers of adherent cells at yolk sac vessels of E17.5 fetuses compared with control stimulation using normal saline (Supplemental Video 2). We also applied TNF-α in E14.5 and E17.5 fetuses and obtained similar numbers of adherent cells as in LPS-stimulated fetuses (Figure 3J). To exclude differences in the expression of TLR4 on the neutrophil surface of E14.5 versus E17.5 fetuses, we performed flow cytometry analysis, which revealed similar expression of TLR4 on neutrophils from E14.5 and E17.5 fetuses compared to neutrophils from adult mice (Supplemental Figure 2E).

Adhesion to inflamed vessels critically depends on the interaction between endothelial ICAM-1 and LFA1 (integrin α_L_β_2_), expressed on neutrophils. Thus, we analyzed the capacity of E14.5, E17.5, and adult neutrophils to bind soluble recombinant murine ICAM-1 (rmICAM-1 hFC chimera) in vitro. As expected, stimulation of neutrophils with the chemokine CXCL1 or using PMA induced an increase in ICAM-1 binding due to LFA1 activation in adult neutrophils, but this effect was severely reduced in neutrophils from E14.5 or E17.5 fetuses (Supplemental Figure 2F). Finally, stimulation of isolated murine neutrophils with TNF-α was unable to induce upregulation of Mac-1 (α_M_β_2_, CD11b/CD18) surface levels in fetal neutrophils (E14.5 and E17.5) to the same extent as in adult neutrophils (Figure 3K).

### A20 upregulation in fetal neutrophils results in diminished adhesion.

**Figure 4:**
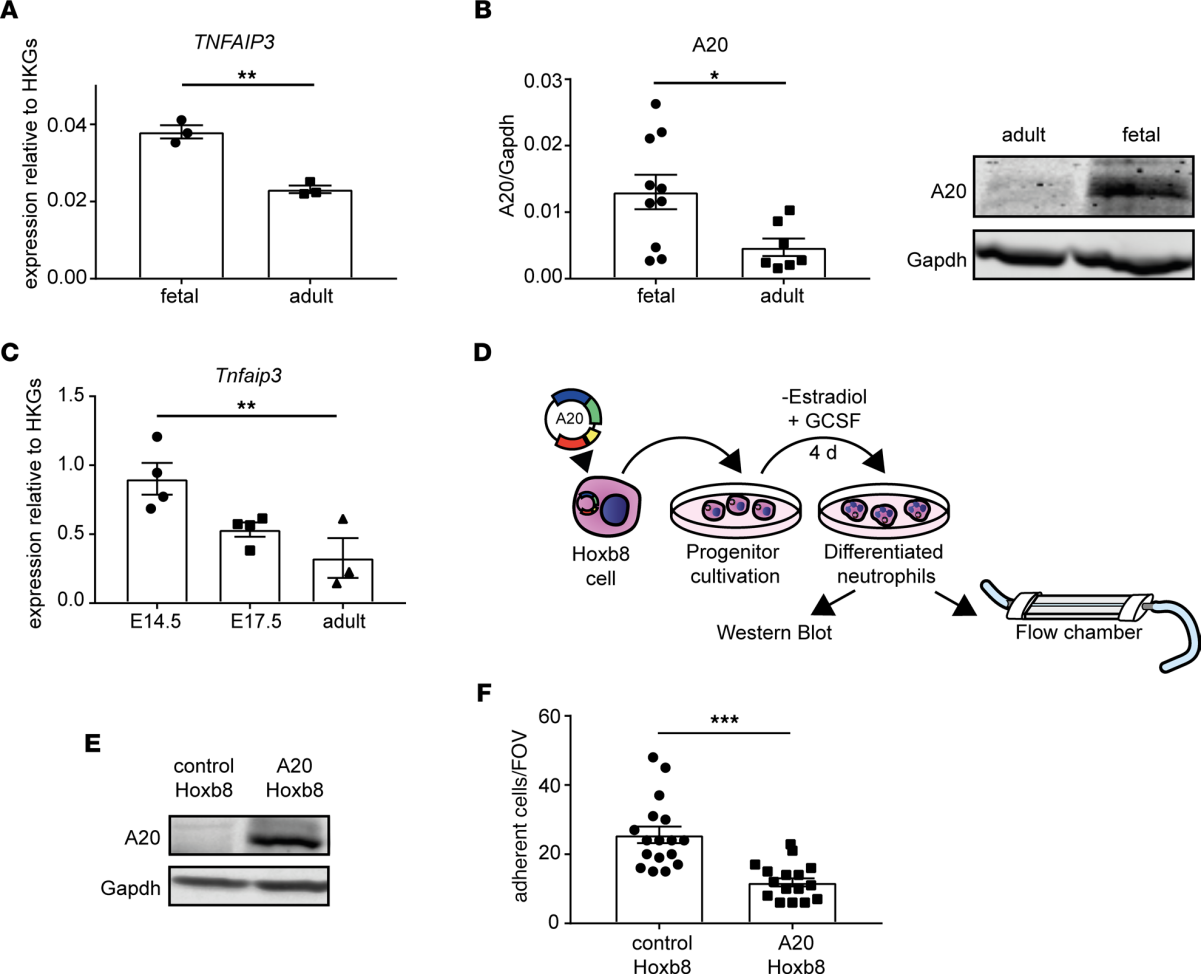
A20 upregulation in fetal neutrophils results in diminished adhesion. (**A**) mRNA expression of *TNFAIP3* in fetal human neutrophils was compared to neutrophils isolated from peripheral blood of adult healthy donors by quantitative RT-PCR. Expression is shown relative to the housekeeping gene (HKG) *GAPDH*. (**P < 0.005; *n* = 3.) Unpaired Student’s *t* test. (**B**) Western blot and respective quantitative analysis of A20 expression in neutrophils isolated from human fetal cord blood samples and neutrophils from adult peripheral blood. Band intensity was normalized to Gapdh protein. All data are presented as mean ± SEM. (*P < 0.05, *n* = 8–10.) Unpaired Student’s *t* test. (**C**) Expression of *Tnfaip3* mRNA relative to the HKGs *B2m* and *Gyk* in murine neutrophils was investigated by quantitative RT-PCR in isolated neutrophils of E14.5 and E17.5 embryos and from the peripheral blood of adult mice. (**P < 0.005; *n* = 3–4.) Ordinary 1-way ANOVA with Dunnett’s multiple comparisons test. (**D**) Workflow of Hoxb8 experiments. Hoxb8 precursor cells overexpressing A20 and Hoxb8 control cells were differentiated into Hoxb8 neutrophils, and subsequently flow chamber experiments were performed in addition to Western blot verification of A20 overexpression. (**E**) Representative Western blot image of A20 expression in control and A20-overexpressing Hoxb8 cells. Gapdh expression is displayed to ensure equal loading. (**F**) Flow chamber analysis of differentiated Hoxb8 control and A20-overexpressing cell adhesion in microflow chambers coated with rmE selectin/rmICAM-1 and rmCXCL1. One representative field was recorded for 10 minutes using an Olympus BX51WI microscope with a CCD camera (model CF8/1, Kappa) and a water immersion objective (×40/0.8 NA, Olympus). All data are presented as mean ± SEM. (***P < 0.001; *n* = 3.) Unpaired Student’s *t* test.

So far, our results revealed that fetal neutrophils exhibit a pronounced baseline activation of the noncanonical NF-κB signaling pathway. Several of the RelB target genes identified in the transcriptomic analysis have immunomodulatory functions and are able to suppress the inflammatory canonical NF-κB signaling pathway. One of those upregulated RelB target genes in fetal neutrophils and detected in the transcriptomic analysis was the ubiquitin-modifying enzyme A20, which has been reported as key negative regulator of innate immune responses. A20 might therefore be an interesting candidate in downmodulating innate immune responses during fetal life. To test this, we first validated A20 upregulation in fetal human neutrophils by real-time PCR (RT-PCR) (Figure 4A) and additionally on the protein level in comparison with adult neutrophils (Figure 4B). We found a significant upregulation of A20 on the transcriptional and on the protein level in fetal neutrophils. Next, we investigated A20 levels in the mouse and validated our findings in human neutrophils, with increased levels of A20 mRNA in fetal versus adult mouse neutrophils (Figure 4C). Interestingly, A20 levels gradually started to decrease at late embryonic stages (E14.5 versus E17.5), which is in line with our in vivo findings of increased adhesion after LPS or TNF-α stimulation at E17.5 versus E14.5. Thus, we conclude that through downregulating A20 expression later during fetal ontogeny, neutrophil adhesiveness increases at the same time. To strengthen this hypothesis, we generated A20-overexpressing Hoxb8 cells and analyzed the adhesive behavior of these cells in a flow chamber system (Figure 4D). Vector-transfected cells without the A20 coding region served as controls. Differentiated Hoxb8 cells have been used as an in vitro and in vivo model system to investigate neutrophil function in mice (24). Western blot analysis of A20-overexpressing differentiated Hoxb8 cells clearly showed a strong upregulation of A20 protein compared with control cells (Figure 4E). To test whether overexpression of A20 influences the adhesive behavior of differentiated Hoxb8 cells, we coated microflow chambers with rmE selectin, rmICAM-1, and rmCXCL1 to mimic the inflamed endothelium and introduced differentiated A20-overexpressing or differentiated control Hoxb8 cells into the chambers at a defined shear rate (1 dyne/cm^2^). Analysis of the number of adherent cells/field of view (FOV) revealed a significant decrease in adhesion of A20-overexpressing cells (Figure 4F), suggesting that A20 is a negative regulator of neutrophil adhesion.

### Increased neutrophil recruitment in Tnfaip3^fl/fl^ Ly6g-Cre mice in vivo.

**Figure 5:**
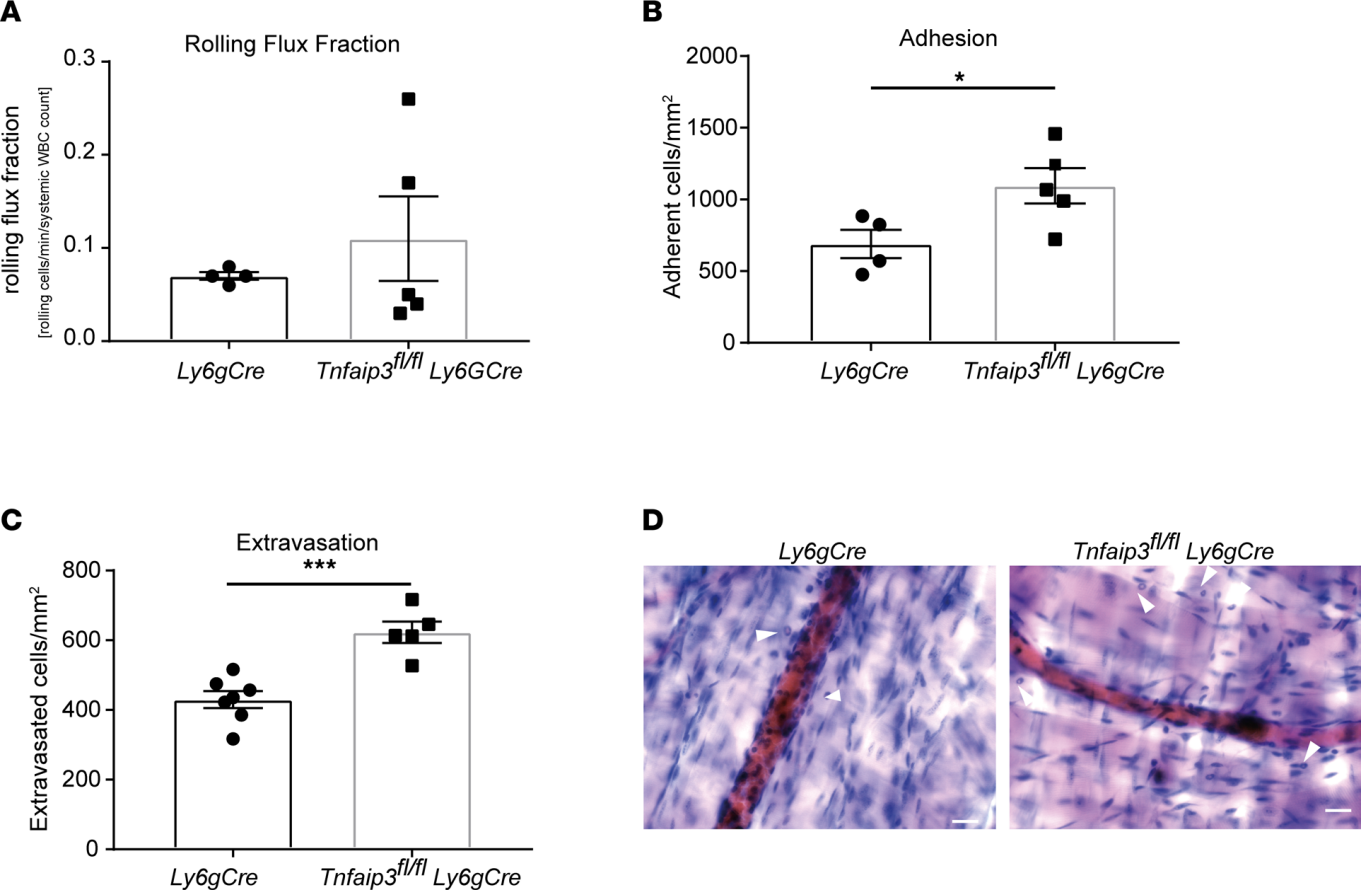
Increased neutrophil recruitment in *Tnfaip3^fl/fl^* *Ly6g-Cre* mice in vivo. In vivo leukocyte rolling, adhesion, and extravasation were analyzed in 2-hour TNF-α–stimulated venules of mouse cremaster muscles in *Ly6g-Cre* and *Tnfaip3^fl/fl^* *Ly6g-Cre* mice. (**A**) Rolling flux fraction (rolling cells/min divided by the total leukocyte flux), (**B**) adherent cells/mm^2^, and (**C**) leukocyte extravasation in the perivascular region of Giemsa-stained cremaster muscle whole mounts. A total of 4 *Ly6g-Cre* (17 vessels) and 5 *Tnfaip3^fl/fl^* *Ly6g-Cre* (17 vessels) mice were analyzed. Values are given as mean ± SEM. (*P < 0.05, ***P < 0.001.) Unpaired Student’s *t* test. (**D**) Representative images of Giemsa-stained whole mounts of cremaster muscles of *Ly6g-Cre* and *Tnfaip3^fl/fl^* *Ly6g-Cre* mice. Arrows point to extravasated neutrophils. Scale bar: 30 μm.

In the next set of experiments, we investigated A20-depleted neutrophils in an in vivo setting of sterile inflammation. Previous observations on A20-depleted immune cells described hyperinflammation in different endpoint mouse models, but so far, the impact of A20 deficiency on the leukocyte adhesion cascade itself is not known. As the A20 constitutive knockout is prematurely lethal (19), we bred *Tnfaip3^fl/fl^* mice to *Ly6g-tdTomato-Cre* mice (*Tnfaip3^fl/fl^* *Ly6g-Cre*), to obtain mice with a highly specific deletion of A20 in mature neutrophils. We first verified A20 depletion in our model and observed a strong decrease in A20 mRNA in neutrophils derived from *Tnfaip3^fl/fl^ Ly6g-Cre* mice, although a complete knockout was not achieved (Supplemental Figure 3A). Using intravital microscopy of TNF-α–stimulated cremaster muscle, we found no differences in the numbers of rolling neutrophils in *Tnfaip3^fl/fl^* *Ly6g-Cre* mice after normalization to the white blood cell count (WBC) compared to *Ly6g-Cre* mice (Figure 5A and Supplemental Video 3). Interestingly, neutrophil rolling velocity was not altered between the groups (Supplemental Figure 3B). Next, we analyzed neutrophil adhesion and found a significant increase in absolute numbers of adherent neutrophils/mm^2^ in *Tnfaip3^fl/fl^* *Ly6g-Cre* mice compared with the control group (Figure 5B), suggesting that loss of A20 in neutrophils leads to a hyperreactive phenotype with increased adhesion to inflamed microvessels. Finally, we stained the exteriorized and fixed cremaster muscle tissue with Giemsa to visualize extravasated neutrophils. Again, we detected a 30% increase of extravasated neutrophils in *Tnfaip3^fl/fl^* *Ly6g-Cre* mice compared with *Ly6g-Cre* mice (Figure 5, C and D). The hemodynamic parameters of both groups did not differ (Table 1). In a second set of in vivo experiments using the cremaster muscle model, experiments were performed within 45 minutes after surgical preparation of the cremaster muscle without additional stimulation. In this mild inflammation model, neutrophil-endothelium interactions are mostly limited to P selectin–dependent rolling, with a few adherent neutrophils (25). Again, hemodynamic parameters were equal in *Tnfaip3^fl/fl^* *Ly6g-Cre* mice compared to *Ly6g-Cre* mice (Table 2). Interestingly, we observed decreased rolling velocities along with increased adhesion of A20-deficient neutrophils compared to *Ly6g-Cre* controls (Supplemental Figure 3, C and D), suggesting a neutrophil-intrinsic hyperreactive phenotype in the absence of A20, which is the contrary effect of what we observed in fetal neutrophils with high A20 expression.

## Discussion

In this study, we aimed to decipher the molecular mechanisms behind the ontogenetic regulation of neutrophil function during fetal development. In a whole transcriptomic survey, we identified over 120 differentially regulated genes in fetal versus adult human neutrophils. Many of those genes upregulated in fetal samples are RelB target genes that together with elevated nuclear RelB localization and higher p52 values suggest a constitutively active noncanonical NF-κB signaling pathway in fetal neutrophils. In contrast, we observed reduced canonical NF-κB signaling in fetal neutrophils upon stimulation and diminished activation of neutrophil adhesion in an intravital microscopy model of the mouse yolk sac of E14.5 fetuses upon intrauterine LPS or TNF-α stimulation. Interestingly, among upregulated genes in fetal neutrophils, we found the ubiquitin-modifying enzyme A20 that is a well-established and potent negative regulator of the inflammatory canonical NF-κB pathway. Indeed, we generated Hoxb8 cells and showed that overexpressing A20 mimics fetal neutrophils and their inability to properly interact with immobilized adhesion molecules in a flow chamber system. On the contrary, murine neutrophils lacking A20 showed a hyperinflammatory phenotype with increased adhesion to inflamed microvessels and extravasation into inflamed cremaster muscle tissue.

Neutrophils are among the body’s first line of defense against invading pathogens. Thus, proper function of neutrophils is critical to reestablish homeostasis without harming the body. Prematurely born infants, especially those born under 33 weeks of gestation, harbor a great risk of infections or sepsis (26). This enhanced susceptibility has been linked to lacking maternal antibodies (27) but also to immature innate immune system compared with term infants (28). It has been shown that, under artificial shear stress, neutrophils from mature born infants demonstrate reduced adhesion compared with adult neutrophils (29), an effect that is even more pronounced in premature neutrophils (7), suggesting an ontogenetic regulation of neutrophil function. Also on the level of direct host defense, neonatal neutrophils lack functionality, as shown by reduced neutrophil extracellular trap formation capability in neonates (30, 31). Interestingly, the molecular mechanisms regulating this ontogenetic maturation are still incompletely understood (28). Using a systems biology approach, a recent study by the Kollmann group investigated peripheral blood samples of newborn infants during the first week of life (9). They performed transcriptomic, proteomic, and metabolomic analyses and revealed a dynamic developmental trajectory affecting the interferon and complement pathway. In addition, the analysis also found changes in neutrophil-associated signaling processes affecting TLR-2 and -9 as well as IL-1–dependent signaling (9). In another recent study, Olin and colleagues investigated immune cell populations and selected plasma proteins during the first 3 months of life in preterm and term infants using mass cytometry (10). The authors identified a rather stereotypic postnatal development of the immune system along one shared trajectory. Interestingly, the authors found a complete segregation of 267 investigated plasma proteins and detected significantly lower neutrophil numbers at birth between preterm and term infants (10). Furthermore, they uncovered upregulation of IFN-γ and CXCL8 production with increasing maturation. Of note, cord blood measurements of the immune system did not correlate well to the immune system status postnatally, which the authors traced back to multifactorial perinatal changes.

To shed new light on the regulation of neutrophils’ function particularly during human fetal ontogeny, we performed a whole-transcriptome approach. To do this, we analyzed neutrophils derived from cord blood of premature and mature infants and compared it to blood-isolated neutrophils from adults, in search for genes that might explain the observed functional difference between fetal and adult neutrophils. Out of 124 differentially regulated genes, we found a prominent cluster of 64 genes that are associated with the NF-κB/RelB pathway (15). Furthermore, our results show higher nuclear RelB levels in fetal samples as well as higher p52 values, indicating an upregulation of the noncanonical NF-κB pathway. Unlike the canonical, inflammatory NF-κB pathway, noncanonical signaling is more diverse, ranging from secondary lymphatic tissue development to circadian rhythm, but also acts as a modulator of immune response (13). While the role of RelB in the myeloid compartment is less known, studies on RelB single- and RelB/p50 double-knockout mice revealed that RelB also represses excessive neutrophil recruitment (32, 33). Those findings suggest that an upregulation of RelB target genes in neonatal neutrophils could act as immune suppressors of the canonical NF-κB pathway. Our results further lead to the assumption that elevated factors in fetal serum like LT-α and an accompanying stronger surface expression of the TNFRII receptor could initiate this increased signaling. Monocytes from preterm infants were shown to display diminished TLR4 expression and MyD88 signaling, along with decreased cytokine production (34, 35). Similar observations were made for neutrophils derived from premature infants (36). Also in our experiments, TNF-α signaling via TNFR led to decreased IκB phosphorylation that would be required to initiate p65 nuclear translocation. This corroborates our hypothesis of a reduced canonical NF-κB signaling in fetal neutrophils as a consequence of immunosuppressive factors transcribed by the RelB pathway. We additionally show that stimulating adult neutrophils with fetal serum for 2 hours markedly inhibited IκB phosphorylation and, at the same time, elevated p52 levels, as an indicator of increased noncanonical signaling. This silencing of myeloid cells observed in our study is supported by a recent report demonstrating that under baseline conditions, TLR4-induced IL-1β production as well as the biological relevant secretion of cleaved IL-1β is diminished in neonatal monocytes due to impaired formation of the NLRP3 inflammasome protein complex (37). Considering the fact that pathologically high levels of cord blood IL-1β are linked to organ damage in the fetus/newborn (38), tuning down innate immune cell function appears to be beneficial for normal fetal development in its physiological environment. We further verified this by intravital imaging of neutrophil recruitment in yolk sac vessels, where we demonstrated an ontogenetic regulation of neutrophil adhesiveness local LPS or TNF-α stimulation.

Among our RelB target genes that were ontogenetically upregulated in fetal neutrophils, we found the ubiquitin-modifying enzyme A20, a complex regulator of NF-κB signaling. Due to its structure, A20 is able to cleave ubiquitin chains, while at the same time acting as a ubiquitin E3 ligase and binding ubiquitin chains. Therefore, A20 interferes with and modifies ubiquitylated proteins and by this mechanism regulates NF-κB signaling in multiple ways. Originally, A20 was described to be activated by the canonical pathway itself (39) and is now well accepted as a negative feedback regulator that silences the initiated inflammatory response (40, 41) and at the same time activates noncanonical NF-κB signaling (42). In A20-overexpressing HEK293 cells, TNF-induced NF-κB activation is abolished (40) in the same fashion as we observed in fetal samples that harbor high A20 expression. To show the functional consequences of high A20 protein levels on immune cell function, we generated A20-overexpressing Hoxb8 cells and observed a decreased adhesion in a flow chamber system. This further strengthens the suggested role of A20 as a key factor that switches fetal neutrophils toward the noncanonical NF-κB pathway and keeps the canonical NF-κB pathway constitutively silent. On the other hand, loss of A20 function due to truncated gene variants or SNPs is associated with auto-inflammatory disorders like Crohn’s disease, psoriasis, rheumatoid arthritis, and many more (16, 43–46). This can also be observed in mice, either with constitutive A20 depletion (19) or with conditional deletion in the hematopoietic compartment (20, 47–50). Interestingly, those mice also display increased IL-1 secretion and increased basal and LPS-induced expression levels of the inflammasome adapter NLRP3 (47, 51). Those previous findings of hyperinflammation in various disease models led us to investigate the neutrophil recruitment cascade itself in the absence of A20, anticipating here an overactive inflammatory phenotype. As expected, we observed more adhesion and transmigration of A20-depleted neutrophils compared with control animals.

Taken together, we provide mechanistic insights into the ontogenetic regulation of neutrophils during fetal life and uncover a shift in NF-κB signaling toward the antiinflammatory noncanonical NF-κB pathway that is accompanied by transcriptional upregulation of A20, a powerful negative regulator of the inflammatory response. In consequence, fetal neutrophils are restricted in their reactivity toward inflammatory signals that would finally lead to neutrophil adhesion and transmigration into inflamed tissue. While this seems to be highly beneficial for the fetus within its protected intrauterine environment, it leads to a substantial increase in morbidity and mortality in prematurely born infants facing the outside world with all its challenges and threats.

## Methods

### Sample collection and study population.

All cord blood samples used in the study were obtained from neonates delivered by Cesarean section at Dr. von Hauner Children’s Hospital or University Hospital Grosshadern, LMU Munich. Immediately after delivery, blood was taken in citrate buffer–containing collection tubes or serum-collecting tubes (S-Monovette, Sarstedt). Exclusion criteria included congenital malformations, known infections of the mother, and familial immune diseases. Blood from adult healthy donors was taken by venipuncture. Blood sampling from adults took place at the Biomedical Center Munich, LMU Munich.

### Microarray and data processing.

Human fetal and adult neutrophils were purified, and RNA was isolated as described below. RNA was loaded on an Hgu133plus (Affymetrix). The R Bioconductor package oligo (52) was used to create expression sets, perform the background correction and quantile normalization per sample, as well as log-transform the data. The microarray data have been deposited in the National Center for Biotechnology Information’s public database Gene Expression Omnibus (accession number: GSE222156). See Table 3 for gestational ages and corresponding cell numbers. We tested all genes on the array for differential expression between the fetal and the adult samples using a Student’s 2-tailed *t* test for each gene separately and then applied the Benjamini-Hochberg procedure to adjust for multiple-hypothesis testing. Probe sets were annotated to Ensembl gene IDs, and summaries are given on the level of Ensembl gene IDs. Differentially expressed genes were defined as all genes with FDR < 5%.

### Gene set enrichment analysis.

Gene set enrichment analysis was performed using WebGestalt (53, 54). All differentially expressed genes (FDR < 5%) were used as input. All genes on the Hgu133plus array were used as background. The minimal gene set size was set to 2. Analyses were run separately for gene sets from the “biological process” and “molecular function” ontologies from GO. The hypergeometric test was used to assess the significance of the overlap of the differentially expressed genes with each of the gene sets. The Benjamini-Hochberg procedure was applied to adjust for multiple-hypothesis testing. Significant gene sets were defined as gene sets with FDR < 5%. None of the gene sets of the “molecular function” ontology was significant.

### Identification of RelB target genes.

We obtained ChIP-Seq peak calls of canonical and noncanonical subunits of Nκ-KB (p65, p50, RelB, and p52) measured in the lymphoblastoid cell line GM12878 (15). For each subunit we defined genes as bound if a binding site of the subunit localized within 1 kb of the gene. Using Fisher’s exact test, we assessed whether binding sites for each subunit were overrepresented or depleted separately in the set of up- and downregulated genes identified by the microarray analysis. Genes that were both differentially expressed and bound by a subunit were defined as target genes of the subunit.

### Mice.

C57BL/6 animals were purchased from Charles River Laboratories and housed in the animal facility of the Biomedical Center Munich at least 1 week before use in experiments. *Lyz2^GFP^*, *Tnfaip3^fl/fl^*, and *Ly6g-Cre* tdTomato (23, 49, 55, 56) mice were generated as described before.

### Generation of A20-overexpressing Hoxb8 cells and differentiation into neutrophil-like cells.

The murine *pWPI*-*A20-IRES-EGFP* vector was a gift (University of California, San Francisco, San Francisco, California, USA). The *pMSCV-Puro* and *pCL-Eco* vectors were provided by Hans Häcker (St. Jude Children’s Research Hospital, Memphis, Tennessee, USA). For viral transduction, the coding region of *A20-IRES-EGFP* was subcloned into the retroviral backbone *pMSCV-Puro*.

For virus production, HEK293T cells (ATCC CRL‑11268) were transfected with *pMSCV-A20-IRES-EGFP* and *pCL-Eco* using Lipofectamine 2000 (Thermo Fisher Scientific) according to the manufacturer’s protocol. Virus-containing supernatant was harvested 48 hours posttransfection, and Hoxb8-SCF cells, generated from C57BL/6 mice as described previously (57, 58), were transduced by spinocculation using Lipofectamine. Upon 72 hours posttransduction, puromycin-resistant cells were selected for the length of 7 days.

Differentiation of Hoxb8-SCF cells toward neutrophils was allowed by culture for 4 days in differentiation medium consisting of RPMI 1640 supplemented with 10% FCS, 1% penicillin/streptomycin, 20 ng/mL rmG-CSF (Peprotech), and 2% SCF-containing supernatant as described (59).

### Purification of human and murine neutrophils.

Human neutrophils were isolated from umbilical cord or peripheral venous blood by layering cells on a Polymorphprep density gradient (AXIS-SHIELD PoC AS) followed by centrifugation (500*g*, 30 minutes, room temperature). The neutrophil layer was harvested, then washed with Dulbecco’s PBS, and erythrocytes were lysed with ammonium chloride lysis buffer. Subsequently, neutrophils were purified by EasySep Human Neutrophil Enrichment Kit (STEMCELL Technologies). Alternatively, the EasySep Direct Human Neutrophil Isolation Kit (STEMCELL Technologies) was used. For quantitative RT-PCR analysis, purity was investigated by flow cytometry and required to be more than 90%.

Murine neutrophils were isolated from fetal blood by decapitation of C57BL/6 fetuses on E13.5/ E14.5 as well as E17.5/E18.5. Blood was layered on a Percoll density gradient (MilliporeSigma). The neutrophil layer was isolated, then washed with Dulbecco’s PBS, and erythrocytes were lysed with ammonium chloride lysis buffer. To yield a highly pure cell population, neutrophils were further purified using the anti-mouse Ly6G–Pacific Blue antibody (BioLegend catalog 127612) by FACS, using a BD FACSAria III instrument.

### RNA processing and quantitative RT-PCR.

RNA of neutrophils (>90% purity) was extracted with the RNeasy Mini Kit (QIAGEN). RNA concentration was measured with a NanoDrop instrument (Thermo Fisher Scientific). Reverse transcription was conducted with High-Capacity cDNA Reverse Transcription Kit (Applied Biosystems), and quantitative RT-PCR was done using TaqMan Gene Expression Assays according to the manufacturer’s protocol (Applied Biosystems). Expression levels were calculated relative to housekeeping genes. See Table 4 for further detail.

### Western blot.

Isolated human and murine neutrophils were homogenized in protein lysis buffer (150 mM NaCl, 1% Triton X-100) (AppliChem), 0.5% Na deoxycholate (MilliporeSigma), 50 mM Tris-HCl pH 7.3 (Merck), and 2 mM EDTA (Merck) supplemented with protease (Roche) and phosphatase inhibitors (MilliporeSigma), and proteins were resolved by SDS-PAGE and then electrophoretically transferred from the gels onto PVDF membranes, which were subsequently blocked in LI-COR blocking solution and incubated with antibodies. The following antibodies were used for detection: mouse/rabbit anti-A20 (Abcam catalog ab13597 and Santa Cruz Biotechnology catalog sc-166692), rabbit anti-p52 (Cell Signaling Technology catalog 4882S), rabbit anti-pIκB (catalog 2859) and rabbit anti-IκB (catalog 4814) (Cell Signaling Technology), and mouse anti-GAPDH (Calbiochem, catalog MAB374). IRDye 680RD (catalog 926-68070) and IRDye800CW (catalog 925-32210) secondary antibodies were purchased from LI-COR. Western blots were scanned using the Odyssey CLx Imaging System and analyzed with Image Studio software (both LI-COR).

### Imaging flow cytometry.

Human cord blood and peripheral blood neutrophils were isolated using the EasySep Direct Human Neutrophil Isolation Kit (STEMCELL Technologies). Afterward, cells were fixed and permeabilized with the Foxp3 Transcription Factor Staining Buffer Set (Affymetrix) according to the manufacturer’s protocol. Intracellular NF-κB was stained with the following antibodies: mouse anti-p65 (F-6) (Santa Cruz Biotechnology catalog sc-8008 AF488) and mouse anti-RelB (Santa Cruz Biotechnology catalog sc-48366 AF488), both conjugated with Alexa Fluor 488. DAPI (Invitrogen) was used to stain the nucleus. Images were acquired using an Amnis ImageStream multispectral imaging flow cytometer. Image analysis was done using Image Data Exploration and Analysis Software (Amnis), applying the nuclear localization wizard. Similarity score defines the overlap of nuclear DAPI signal and NF-κB signal.

### Flow cytometry.

Surface expression of TLR4 (anti-mouse TLR4-PE, BioLegend, catalog 145403), LTβR (anti-mouse catalog 134403 or anti-human catalog 322008 LTβR-PE, BioLegend), TNFRI (TNFRI-APC, BioLegend catalog 369906), or TNFRII (anti-mouse catalog 113406 or anti-human catalog B293743 TNFRII-PE-Cy7, BioLegend) was assessed on neutrophils from human cord or peripheral blood or mouse peripheral blood using a Beckman Coulter Gallios or a Cytoflex S flow cytometer and analyzed with FlowJo Analysis Software. Surface expression of CD11b (Mac-1) (anti–mouse or anti–human Cd11b-AF700 BioLegend catalog 101222) and CD18 (integrin β2 subunit) (anti-human CD18-FITC, BioLegend catalog 302106) was assessed after 2 hours of incubation either with 20 ng/mL recombinant human TNF-α or HBSS as control using Cytoflex S flow cytometer and FlowJo Analysis Software for analysis. Gating of human neutrophils was performed using CD15 (anti-human CD15-APC, BioLegend catalog 323008) and CD66b (anti-human CD66b-PB, BioLegend catalog 305112). For the soluble ICAM-1 binding assay, please refer to Supplemental Methods.

### Flow chamber.

Ibidi flow chambers (0.5 μm Slide VI0.1) were coated with E selectin (rhCD62E-Fc chimera, 5 μg/mL; R&D Systems), ICAM-1 (rhICAM-1, 4 μg/mL; R&D Systems), and CXCL8 (rhCXCL-8, 10 μg/mL, Peprotech) overnight at 4°C. The next day, chambers were blocked with 5% casein. Prior to the start of experiments, isolated human adult neutrophils were incubated for 2 hours with either HBSS (control) or adult or fetal serum, washed twice, and then diluted in HBSS to 1 × 10^6^ cells/mL. Perfusion through the flow chamber was conducted with a high-precision pump (Harvard Apparatus) at a shear stress level of 1 dyne/cm^2^. Experiments were conducted on a ZEISS AXIOVERT 200 microscope, provided with a ZEISS LD Plan-neofluor objective (20×, 0.4 NA: and a SPOT RT ST Camera). MetaMorph software was used to generate movies for later analysis using Fiji software.

### Phagocytosis.

Human peripheral blood neutrophils were isolated using the EasySep Direct Human Neutrophil Isolation Kit (STEMCELL Technologies). Cells were stimulated for 2 hours with either RPMI (control) or fetal or adult serum, washed 2 times, afterward incubated with Zymosan particles for another 2 hours, and further processed as described in the data sheet (Phagocytosis Assay Kit, Green Zymosan). Samples were analyzed in the FL1 channel of a Cytoflex S flow cytometer and data analyzed by FlowJo Analysis Software.

### LT-α ELISA.

Serum was obtained from human cord and adult peripheral blood. The assay was performed according to the manufacturer’s instructions (R&D Systems). Samples were run in duplicates and analyzed using a microplate reader (TECAN).

### Surgical preparation of the yolk sac and intravital microscopy.

Pregnant *Lyz2^GFP^* mice (E14.5–E17.5) were anesthetized intraperitoneally with 5 mg/mL ketamine and 1 mg/mL xylazine in 10 mL/kg of normal saline. Two hours prior to intravital microscopy, the uterine horn was carefully exteriorized through an abdominal wall incision followed by the intrauterine injection of 100 μL LPS (1 μg/μL in 0.9% NaCl), 50 μL of TNF-α (10 ng/μL in 0.9% NaCl), or NaCl (0.9%). During the intrauterine injection between 2 fetuses, care was taken not to damage the fetuses or their surrounding yolk sacs. The site of injection between 2 fetuses was marked by a small knot using silk braided suture. Thereafter, the uterine horn was returned, and the abdominal wall incision was temporarily closed by a small metallic clamp. After 2 hours, intravital microscopy (La Vision Biotech and Olympus BX51) to analyze leukocyte recruitment was performed as previously described (8).

### Intravital microscopy of TNF-α–stimulated mouse cremaster muscle venules.

Mice were treated by intrascrotal injection of 500 ng TNF-α (R&D Systems) 2 hours prior to microscopy, anesthetized, and prepared for intravital microscopy, as described (60). Movies from cremasteric postcapillary venules ranging from 20 to 40 μm in diameter were recorded using BX51WI microscope (Olympus) with a water immersion objective ×40, 0.80 NA, and an Olympus charge-coupled device camera (CF8/1, Kappa). Blood samples were taken after the experiments, and WBC and neutrophil counts were determined using ProCyte Dx Hematology Analyzer (IDEXX). Rolling velocity and leukocyte adhesion efficiency (number of adherent cells/mm^2^ divided by the systemic neutrophil count) were calculated on the basis of the recorded movies using Fiji software (61). Afterward, cremaster muscles were fixed with 4% PFA (AppliChem) and stained using Giemsa (MilliporeSigma). The number of perivascular cells/mm^2^ was calculated with a Leica DM2500 microscope equipped with a DMC2900 CMOS camera and an HCX PL APO 100×/1.40 Oil Ph3.

### Statistics.

All data were analyzed and plotted using Graph Pad Prism Software. For pairwise comparison of experimental groups, a paired 2-tailed Student’s *t* test was performed; for comparison of independent samples, the unpaired Student’s *t* test was used. Depending on the condition, we used 1-way ANOVA with either Dunnett’s post hoc test (comparison of experimental groups against control) or Tukey’s post hoc test (comparison of all experimental groups against each other) or a 2-way ANOVA with Tukey’s post hoc test (comparison of paired experimental groups against each other) for multiple comparison. *P* values less than 0.05 were considered statistically significant.

### Study approval.

All experiments using human cord blood were approved by the ethics committee of LMU Munich, project 249-08. All animal experiments were approved by the Regierung von Oberbayern, Munich, Germany (AZ 55.2-1-54-2531-80-76/12, 55.2-1-54-2532.102.2017 and 02-18-26).

## Author contributions

IR, LMW, and KH designed and conducted experiments, analyzed data, and wrote the manuscript. AB, JA, MR, SB, and M Salvermoser acquired and analyzed data. CN and TK provided human cord blood samples, and MG, AM, and CS provided mice. MSS and BW provided critical reagents and their expertise. TS, KH, and MH analyzed data. M Sperandio designed experiments and wrote the manuscript.

## Supplementary Material

Supplemental data

Supplemental table 1

Supplemental video 1

Supplemental video 2

Supplemental video 3

## Figures and Tables

**Table 1:**
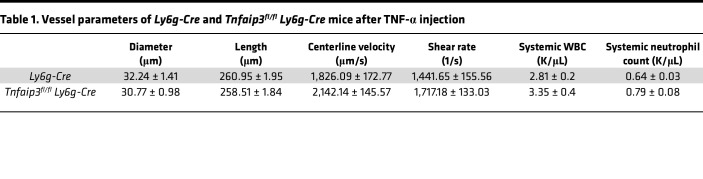
Vessel parameters of *Ly6g-Cre* and *Tnfaip3^fl/fl^ Ly6g-Cre* mice after TNF-α injection

**Table 2:**
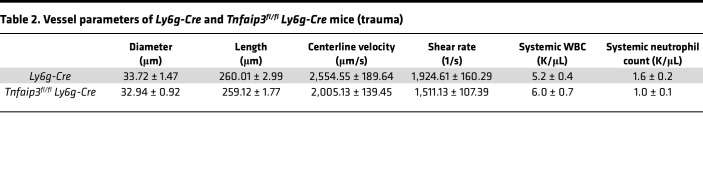
Vessel parameters of *Ly6g-Cre* and *Tnfaip3^fl/fl^* *Ly6g-Cre* mice (trauma)

**Table 3:**
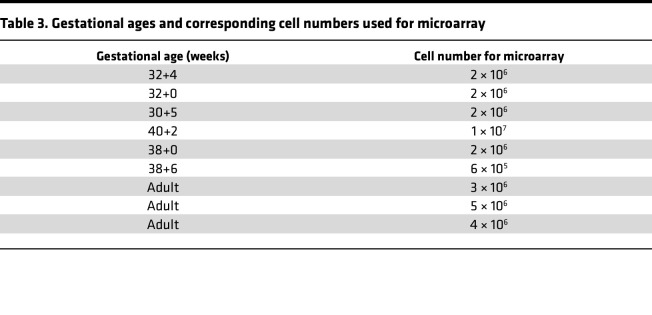
Gestational ages and corresponding cell numbers used for microarray

**Table 4:**
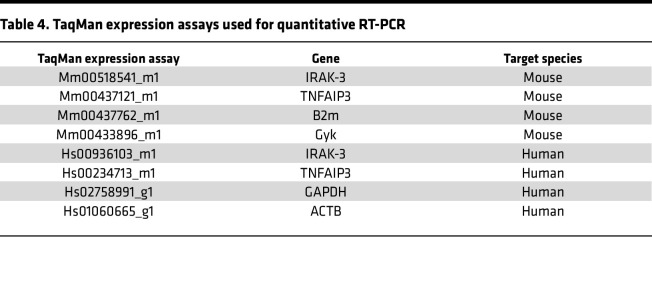
TaqMan expression assays used for quantitative RT-PCR

## Version 1 01/12/2023

In-Press Preview

## Version 2 02/22/2023

Electronic publication

## References

[1] Stoll BJ (2002). Late-onset sepsis in very low birth weight neonates: the experience of the NICHD Neonatal Research Network. Pediatrics.

[2] Liu L (2016). Global, regional, and national causes of under-5 mortality in 2000-15: an updated systematic analysis with implications for the sustainable development goals. Lancet.

[3] Levy O (2007). Innate immunity of the newborn: basic mechanisms and clinical correlates. Nat Rev Immunol.

[4] Henneke P (2021). Perinatal development of innate immune topology. Elife.

[5] Burg ND, Pillinger MH (2001). The neutrophil: function and regulation in innate and humoral immunity. Clin Immunol.

[6] Németh T (2020). Neutrophils as emerging therapeutic targets. Nat Rev Drug Discov.

[7] Nussbaum C (2013). Neutrophil and endothelial adhesive function during human fetal ontogeny. J Leukoc Biol.

[8] Sperandio M (2013). Ontogenetic regulation of leukocyte recruitment in mouse yolk sac vessels. Blood.

[9] Lee AH (2019). Dynamic molecular changes during the first week of human life follow a robust developmental trajectory. Nat Commun.

[10] Olin A (2018). Stereotypic immune system development in newborn children. Cell.

[11] Zhong W (2020). Dramatic changes in blood protein levels during the first week of life in extremely preterm infants. Pediatr Res.

[12] Zhang Q (2017). 30 years of NF-κB: a blossoming of relevance to human pathobiology. Cell.

[13] Millet P (2013). RelB: an outlier in leukocyte biology. J Leukoc Biol.

[14] Yilmaz ZB (2003). RelB is required for Peyer’s patch development: differential regulation of p52-RelB by lymphotoxin and TNF. EMBO J.

[15] Zhao B (2014). The NF-κB genomic landscape in lymphoblastoid B cells. Cell Rep.

[16] Ma A, Malynn BA (2012). A20: linking a complex regulator of ubiquitylation to immunity and human disease. Nat Rev Immunol.

[17] Catrysse L (2014). A20 in inflammation and autoimmunity. Trends Immunol.

[18] Shembade N, Harhaj EW (2012). Regulation of NF-κB signaling by the A20 deubiquitinase. Cell Mol Immunol.

[19] Lee EG (2000). Failure to regulate TNF-induced NF-kappaB and cell death responses in A20-deficient mice. Science.

[20] Matmati M (2011). A20 (TNFAIP3) deficiency in myeloid cells triggers erosive polyarthritis resembling rheumatoid arthritis. Nat Genet.

[21] Blum KS, Pabst R (2006). Keystones in lymph node development. J Anat.

[22] Phillipson M (2006). Intraluminal crawling of neutrophils to emigration sites: a molecularly distinct process from adhesion in the recruitment cascade. J Exp Med.

[23] Faust N (2000). Insertion of enhanced green fluorescent protein into the lysozyme gene creates mice with green fluorescent granulocytes and macrophages. Blood.

[24] Orosz A (2021). In vivo functions of mouse neutrophils derived from HoxB8-transduced conditionally immortalized myeloid progenitors. J Immunol.

[25] Sperandio M (2003). P-selectin glycoprotein ligand-1 mediates L-selectin-dependent leukocyte rolling in venules. J Exp Med.

[26] Kan B (2016). An immunological perspective on neonatal sepsis. Trends Mol Med.

[27] Van Den Berg JP (2010). Transplacental transport of IgG antibodies specific for pertussis, diphtheria, tetanus, haemophilus influenzae type b, and neisseria meningitidis serogroup C is lower in preterm compared with term infants. Pediatr Infect Dis J.

[28] Sharma AA (2012). The developing human preterm neonatal immune system: a case for more research in this area. Clin Immunol.

[29] Mariscalco MM (1998). P-Selectin support of neonatal neutrophil adherence under flow: contribution of L-selectin, LFA-1, and ligand(s) for P-selectin. Blood.

[30] Yost CC (2009). Impaired neutrophil extracellular trap (NET) formation: a novel innate immune deficiency of human neonates. Blood.

[31] Marcos V (2009). Delayed but functional neutrophil extracellular trap formation in neonates. Blood.

[32] Weih F (1995). Multiorgan inflammation and hematopoietic abnormalities in mice with a targeted disruption of RelB, a member of the NF-kappaB/Rel family. Cell.

[33] Weih F (1997). p50-NF-kappaB complexes partially compensate for the absence of RelB: Severely increased pathology in p50(-/-)relB(-/-)double-knockout mice. J Exp Med.

[34] Förster-Waldl E (2005). Monocyte toll-like receptor 4 expression and LPS-induced cytokine production increase during gestational aging. Pediatr Res.

[35] Sadeghi K (2007). Immaturity of infection control in preterm and term newborns is associated with impaired toll-like receptor signaling. J Infect Dis.

[36] Al-Hertani W (2007). Human newborn polymorphonuclear neutrophils exhibit decreased levels of MyD88 and attenuated p38 phosphorylation in response to lipopolysaccharide. Clin Invest Med.

[37] Sharma AA (2015). Impaired NLRP3 inflammasome activity during fetal development regulates IL-1β production in human monocytes. Eur J Immunol.

[38] Dinarello CA (2009). Immunological and inflammatory functions of the interleukin-1 family. Annu Rev Immunol.

[39] Krikos A (1992). Transcriptional activation of the tumor necrosis factor alpha -inducible zinc finger protein, A20, is mediated by kappa B elements. J Biol Chem.

[40] Song HY (1996). The tumor necrosis factor-inducible zinc finger protein A20 interacts with TRAF1/TRAF2 and inhibits NF-kappaB activation. Proc Natl Acad Sci U S A.

[41] Heyninck K, Beyaert R (1999). The cytokine-inducible zinc finger protein A20 inhibits IL-1-induced NF-kappaB activation at the level of TRAF6. FEBS Lett.

[42] Yamaguchi N (2013). Involvement of A20 in the molecular switch that activates the non-canonical NF-κB pathway. Sci Rep.

[43] Vereecke L (2009). The ubiquitin-editing enzyme A20 (TNFAIP3) is a central regulator of immunopathology. Trends Immunol.

[44] Lodolce JP (2010). African-derived genetic polymorphisms in TNFAIP3 mediate risk for autoimmunity. J Immunol.

[45] Elsby LM (2010). Functional evaluation of TNFAIP3 (A20) in rheumatoid arthritis. Clin Exp Rheumatol.

[46] Adrianto I (2011). Association of a functional variant downstream of TNFAIP3 with systemic lupus erythematosus. Nat Genet.

[47] Walle L Vande (2014). Negative regulation of the NLRP3 inflammasome by A20 protects against arthritis. Nature.

[48] Das T (2018). A20/tumor necrosis factor α-induced protein 3 in immune cells controls development of autoinflammation and autoimmunity: lessons from mouse models. Front Immunol.

[49] Tavares RM (2010). The ubiquitin modifying enayme A20 restricts B cell survival and prevents autoimmunity. Immunity.

[50] Hammer GE (2011). Dendritic cell expression of A20 preserves immune homeostasis and prevents colitis and spondyloarthritis Gianna. Nat Immunol.

[51] Duong BH (2015). A20 restricts ubiquitination of pro-interleukin-1β protein complexes and suppresses NLRP3 inflammasome activity. Immunity.

[52] Carvalho BS, Irizarry RA (2010). A framework for oligonucleotide microarray preprocessing. Bioinformatics.

[53] Zhang B (2005). WebGestalt: an integrated system for exploring gene sets in various biological contexts. Nucleic Acids Res.

[54] Wang J (2013). WEB-based GEne SeT AnaLysis Toolkit (WebGestalt): update 2013. Nucleic Acids Res.

[55] Hasenberg A (2015). Catchup: a mouse model for imaging-based tracking and modulation of neutrophil granulocytes. Nat Methods.

[56] Stremmel C (2018). Yolk sac macrophage progenitors traffic to the embryo during defined stages of development. Nat Commun.

[57] Wang GG (2006). Quantitative production of macrophages or neutrophils ex vivo using conditional Hoxb8. Nat Methods.

[58] Redecke V (2013). Hematopoietic progenitor cell lines with myeloid and lymphoid potential. Nat Methods.

[59] Zehrer A (2018). A fundamental role of Myh9 for neutrophil migration in innate immunity. J Immunol.

[60] Pruenster M (2015). Extracellular MRP8/14 is a regulator of β2 integrin-dependent neutrophil slow rolling and adhesion. Nat Commun.

[61] Schindelin J (2012). Fiji: an open-source platform for biological-image analysis. Nat Methods.

