# Supplementary material for: A20 and the noncanonical NF-κB pathway are key regulators of neutrophil recruitment during fetal ontogeny
Source: JCI Insight. 2023 Feb 22;8(4):e155968. doi: 10.1172/jci.insight.155968 (PMC9977499; doi:10.1172/jci.insight.155968)
Supplement: Supplemental data [file jciinsight-8-155968-s018.pdf]

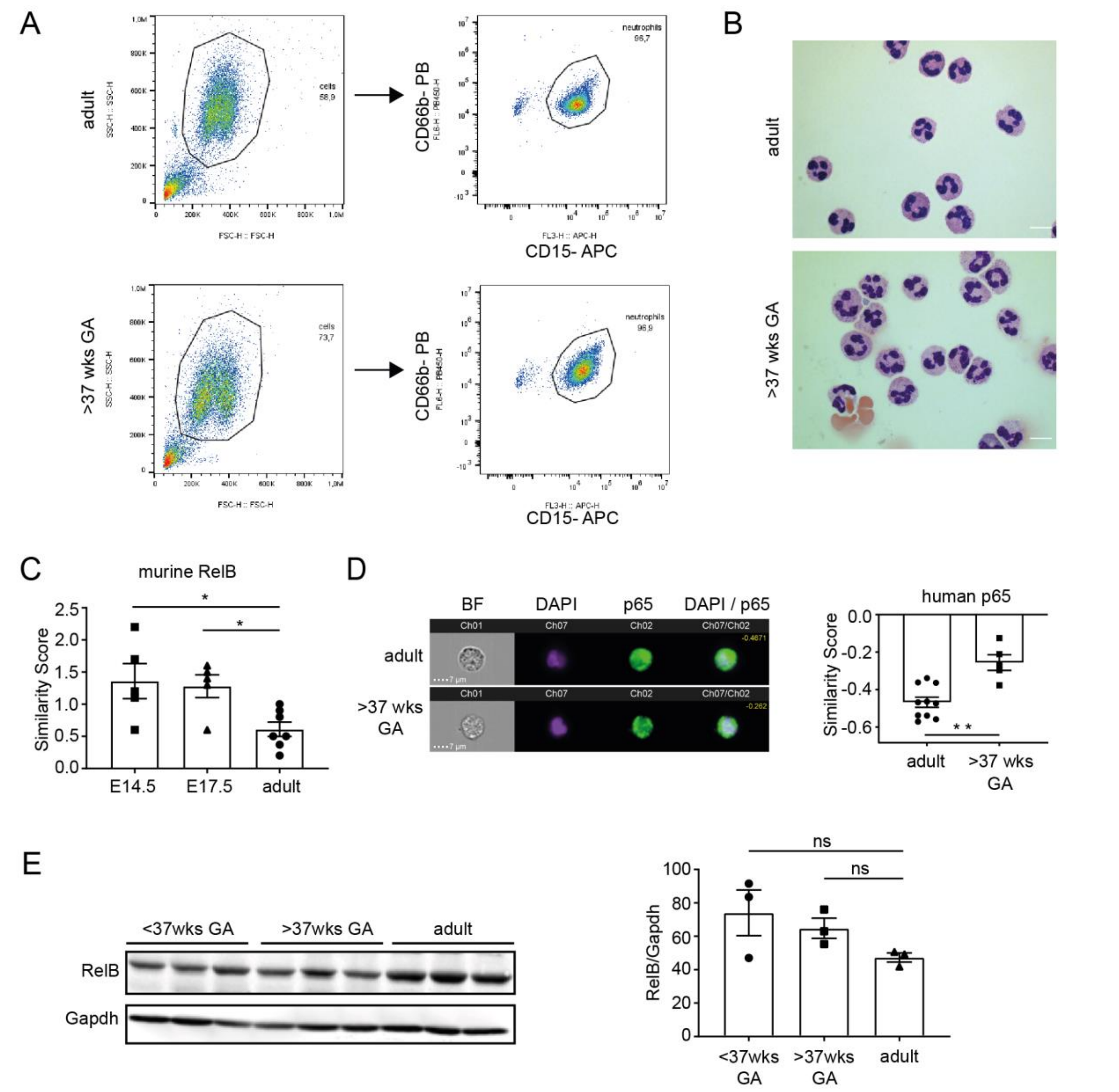

Supplemental Figure 1: Comparison of human adult and fetal neutrophils, RelB and p65 localization as well as RelB protein expression levels.

(A) Representative forward scatter vs. side scatter dot plots of isolated human adult and fetal (gestational age >37 weeks) neutrophils (left panels). Gating of human neutrophils was performed using CD15 and CD66b (right panels). Cytoflex S flow cytometer was used and data was analyzed with the FlowJo Analysis Software. (B) Representative images of May-Gruenwald Giemsa stained cytospin slides of isolated human adult and fetal (gestational age >37 weeks) neutrophils. Images were obtained using a Leica DM2500 microscope equipped with a DMC2900 CMOS camera and a HCX PL APO 100x/1.40 Oil Ph3. Scale bar: 10µm (C) Imaging flow cytometry was performed on murine adult and E14.5 and E17.5 fetal neutrophils and nuclear RelB was quantified. Similarity score defines overlap of nuclear DAPI signal and the respective NF-κB subunit. All data is presented as mean ± SEM. (* $p<0.05$, n= 5-7) Ordinary one-way ANOVA with Tukey's multiple comparisons test. (D) Imaging flow cytometry was performed on cord blood neutrophils from mature infants (gestational age >37 weeks) and peripheral blood from adult healthy donors. Nuclear signal was determined by DAPI and additionally the NF-κB subunit p65 was visualized. Representative pictures of fetal and adult neutrophils are shown as brightfield (BF) image, DAPI, NF-κB subunit p65 and a DAPI/p65 overlay. Respective similarity scores are displayed. Scale bar: 7µm. Similarity score defines overlap of nuclear DAPI signal and the respective

NF-κB subunit. All data is presented as mean ± SEM (** $p<0.005$, n= 5-10). Mann Whitney test. (E) Western blot and respective quantitative analysis of RelB in neutrophils isolated from premature (gestational age (GA) <37 weeks) and mature infants (gestational age >37 weeks) and peripheral blood from healthy adult donors. Band intensity was normalized to Gapdh. All data is presented as mean ± SEM. (ns= not significant; n= 3); Ordinary one-way ANOVA with Dunnett's multiple comparisons test.

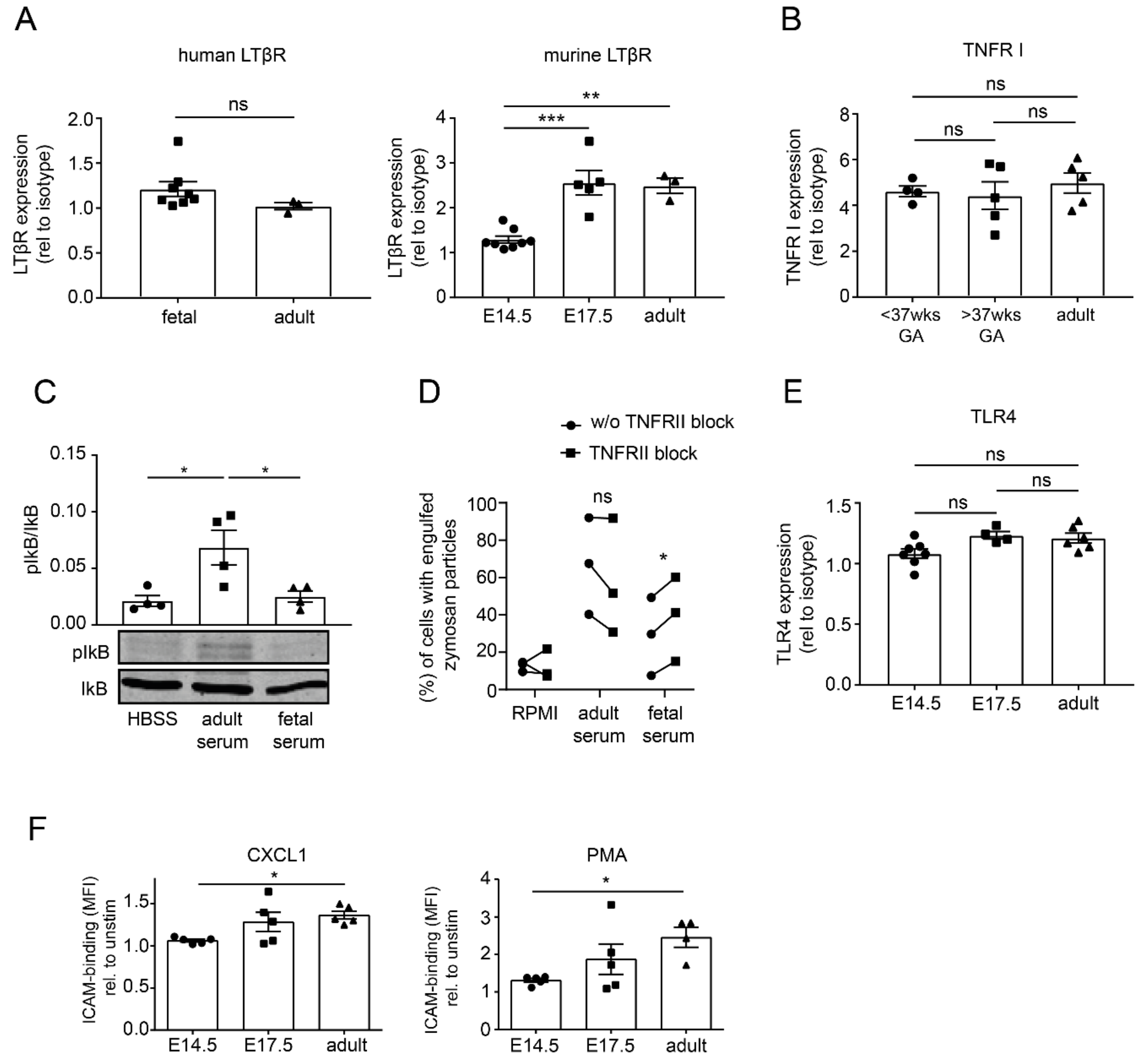

Supplemental Figure 2: Surface marker levels in fetal and adult neutrophils, stimulation of fetal neutrophils with adult and fetal serum as well as ICAM-1 binding assay

(A) Flow cytometry analysis of LTβR expression on human and murine neutrophils out of whole blood from indicated gestational ages. Median fluorescence intensity normalized to isotype control is displayed. All data is presented as mean ± SEM. (ns= not significant, ** p<0.005, *** p<0.001; n= 6). (B) Flow cytometry analysis of TNFR I expression on human neutrophils out of whole blood from indicated gestational ages. Median fluorescence intensity normalized to isotype control is displayed. All data is presented as mean ± SEM. (ns= not significant; n= 4-5/group). Ordinary one-way ANOVA with Dunnett's multiple comparisons test. (C) Western blot and respective quantitative analysis of phospho-IκB in fetal neutrophils after HBSS (control) incubation or stimulation with adult or fetal serum. Band intensities were normalized to total IκB protein. All data is presented as mean ± SEM. (* p<0.05; n= 4) Ordinary one-way ANOVA with Tukey's multiple comparisons test. (D) Quantitative analysis of phagocytosis of human adult neutrophils by measuring the amount of engulfed zymosan particles by flow cytometry after incubation with either RPMI (w/o TNFRII block) or blocking with anti-human TNRFII antibody (TNFRII block) for 2 h prior to stimulation without (RPMI) or with adult or fetal serum. All data is presented as mean ± SEM. (ns= not significant, * p<0.05; n= 3) Paired student´s t-test for each group (RPMI, adult serum and fetal serum). (E) Flow cytometry analysis of

the LPS receptor TLR4 expression on the cell surface of fetal and adult mouse neutrophils. Median expression levels relative to the respective isotype control are displayed. All data is presented as mean ± SEM. (ns = not significant; n=4-7). Ordinary one-way ANOVA with Dunnett's multiple comparisons test. (F) Soluble ICAM-1 binding to LFA-1 on murine neutrophils was investigated in vitro. Values of ICAM-1 binding to unstimulated control cells was set to one and results are shown relative to unstimulated controls. Data are presented as mean +SEM (* $p<0.05$, n=5-7). Ordinary one-way ANOVA with Tukey's multiple comparisons test.

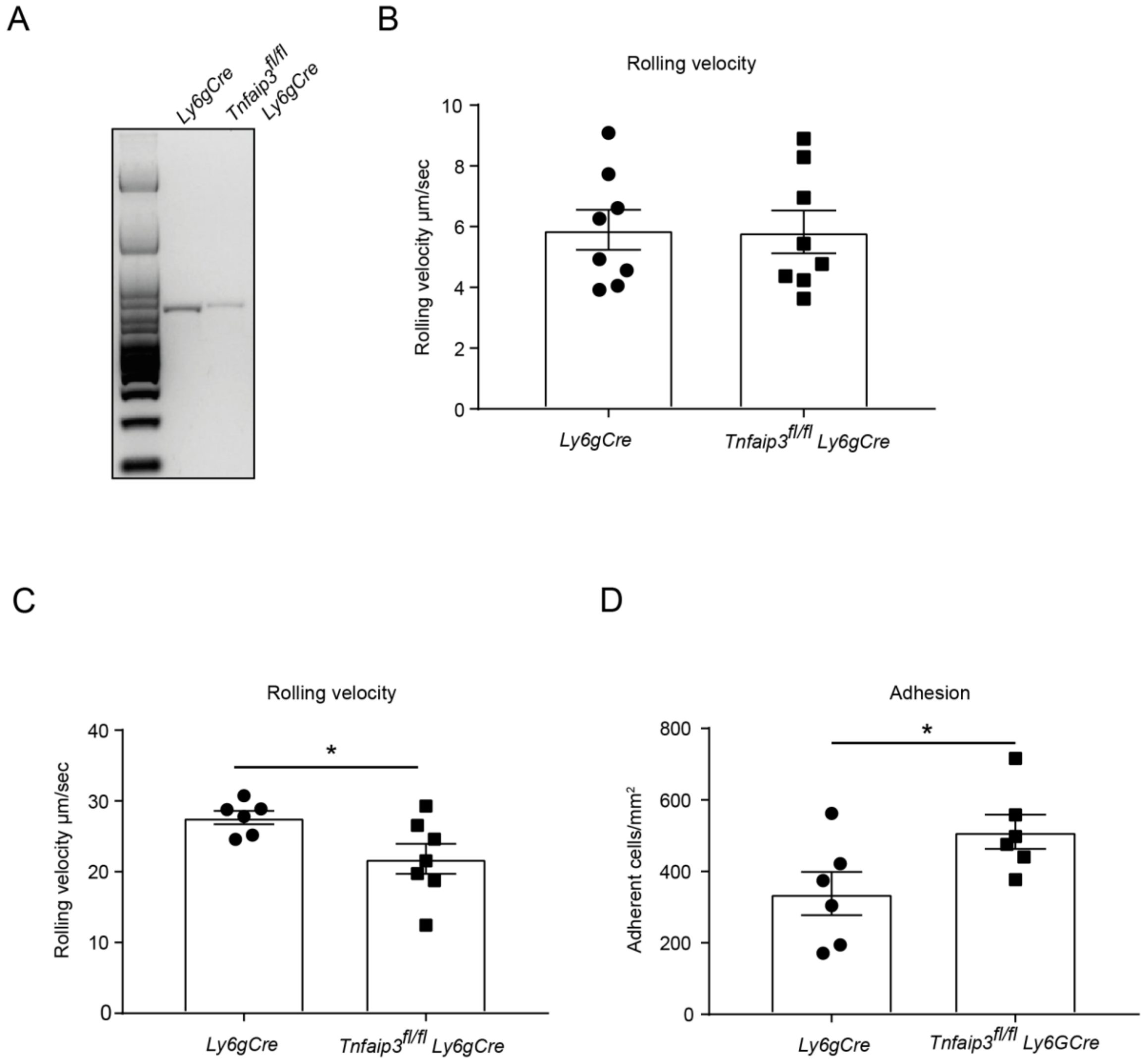

Supplemental Figure 3: Neutrophil adhesion in *Tnfaip3*$^{fl/fl}$ *Ly6gCre* mice.

(A) Representative images of A20 mRNA levels in *Ly6gCre* and *Tnfaip3*$^{fl/fl}$ *Ly6gCre* isolated neutrophils. (B) In vivo leukocyte rolling velocity was analyzed in 2h TNF-α -stimulated venules of mouse cremaster muscles in 8 *Ly6gCre* and 8 *Tnfaip3*$^{fl/fl}$ *Ly6gCre* mice. (C) In vivo leukocyte rolling velocity and (D) leukocyte adhesion was analyzed in surgically prepared venules of mouse cremaster muscles in 6 *Ly6gCre* and 6 *Tnfaip3*$^{fl/fl}$ *Ly6gCre* mice. Values are given as mean ± SEM; * $p<0.05$. Unpaired student´s t-test.

Supplemental Movie 1 and 2: In vivo imaging of E14.5 and E17.5 yolk sac vessels after LPS stimulation
Adhesion of fetal neutrophils at E14.5 (Movie 1) and E17.5 (Movie 2) in yolk sac vessels in vivo was assessed 2h after *Lyz2*^{GFP} mice received an intrauterine injection of 100ng LPS. Using fluorescence microscopy vessels were recorded for approximately 1 minute and neutrophils identified due to their GFP signal. Movies were analyzed using Fiji software. Scale bar: 30µm.

Supplemental Movie 3: In vivo imaging of inflamed cremaster muscle venules of *Tnfaip3*$^{fl/fl}$ *Ly6gCre* and *Ly6gCre* mice
In vivo leukocyte rolling and adhesion was analyzed in 2h TNF-α-stimulated venules of mouse cremaster muscles. Each vessel was recorded for approximately 1 min using BX51WI microscope with a water immersion objective ×40, 0.80 NA and a Olympus CCD camera (CF8/1, Kappa). Movies were analyzed using Fiji software. Scale bar: 30µm.

Supplemental Table 1: Candidate genes according to their function and association with biological processes
WEB-based Gene SeT AnaLysis Toolkit (WebGestalt) was applied, to group candidate genes according to their function and association with biological processes.

Supplemental Table 2: Overrepresentation of RelB subunit in promoters of upregulated genes

				**Not regulated**		**Regulated**	
**Subunit**	**Regulation**	**P-value**	**Odds ratio**	**not bound**	**bound**	**not bound**	**bound**
p65	down	0.00026281	2.82844308	4462	4042	16	41
RelB	down	5.19E-07	4.05754114	5383	3121	17	40
p50	down	5.72E-06	3.6366211	7087	1417	33	24
p52	down	0.00052564	2.54197721	5789	2715	26	31
p65	up	0.28319133	0.75556444	4436	4054	42	29
RelB	up	1	0.98692307	5355	3135	45	26
p50	up	0.00975615	0.29304986	7053	1437	67	4
p52	up	0.37327176	0.77222254	5763	2727	52	19

Supplemental Table 3: Table of RelB- up- and downregulated genes in cord blood neutrophils in comparison to adult neutrophils.

**Upregulated in cord blood neutrophils**	
**Gene symbol**	**Gene name**
JAG1	jagged 1
ALDH2	aldehyde dehydrogenase 2 family (mitochondrial)
ARG1	arginase 1
ENO1	enolase 1, (alpha)
HES1	hairy and enhancer of split 1, (Drosophila)
IGF1R	insulin-like growth factor 1 receptor
IL6ST	interleukin 6 signal transducer (gp130, oncostatin M receptor)
IL10RA	interleukin 10 receptor, alpha
LDLR	low density lipoprotein receptor
MAK	male germ cell-associated kinase
NFE2L2	nuclear factor, erythroid 2-like 2

P4HA1	prolyl 4-hydroxylase, alpha polypeptide I
PPP1CB	protein phosphatase 1, catalytic subunit, beta isozyme
PTPN7	protein tyrosine phosphatase, non-receptor type 7
RALGDS	ral guanine nucleotide dissociation stimulator
SKIL	SKI-like oncogene
SLC8A1	solute carrier family 8 (sodium/calcium exchanger), member 1
TFRC	transferrin receptor (p90, CD71)
KLF10	Kruppel-like factor 10
TNFAIP3	tumor necrosis factor, alpha-induced protein 3
UPP1	uridine phosphorylase 1
EZR	Ezrin
IL1R2	interleukin 1 receptor, type II
SLC7A5	solute carrier family 7 (amino acid transporter light chain, L system), member 5
GAS7	growth arrest-specific 7
DGKD	diacylglycerol kinase, delta 130kDa
BHLHE40	basic helix-loop-helix family, member e40
COX7A2L	cytochrome c oxidase subunit VIIa polypeptide 2 like
SF3A3	splicing factor 3a, subunit 3, 60kDa
IRAK3	interleukin-1 receptor-associated kinase 3
ELL2	elongation factor, RNA polymerase II, 2
ZNF292	zinc finger protein 292
CLIC4	chloride intracellular channel 4
TIAM2	T-cell lymphoma invasion and metastasis 2
RABGEF1	RAB guanine nucleotide exchange factor (GEF) 1
PAG1	phosphoprotein associated with glycosphingolipid microdomains 1
SAMSN1	SAM domain, SH3 domain and nuclear localization signals 1
RILPL2	Rab interacting lysosomal protein-like 2
IRF2BP2	interferon regulatory factor 2 binding protein 2
PIM3	pim-3 oncogene

**Downregulated in cord blood neutrophils**	
**Gene symbol**	**Gene name**
RHOB	ras homolog family member B
ATP6V0A1	ATPase H+ transporting VO subunit a1
CALM3	calmodulin 3
GBP1	guanylate binding protein 1
NFIC	nuclear factor I C
EIF3H	eukaryotic translation initiation factor 3 subunit Hrabaptin, RAB GTP
RABEP1	rabaptin, RAB GTPase binding effector protein 1
MED27	mediator complex subunit 27
VTI1B	vesicle transport through interaction with t-SNAREs 1B
TCFL5	transcription factor like 5
SP140	SP140 nuclear body protein
ZFYVE26	zinc finger FYVE-type containing 26

CCDC28A	coiled-coil domain containig 28A
P2RY10	P2Y receptor family member 10
BAZ2B	bromodomain adjacent to zinc finger domain 2B
TAPBPL	TAP binding protein like
C7orf43	chromosome 7 open reading frame 43
NAGK	N-acetylglucosamine kinase
PNRC2	proline rich nuclear receptor coactivator 2
PRRG4	proline rich and Gla domain 4
ASCC2	activating signal cointegrator 1 complex subunit 2
RAVER1	ribonucleoprotein, PTB binding 1
RPP25L	ribonuclease P/MRP subunit p25 like
MICA	MHC class I polypeptide-related sequence A

Supplemental Methods

Soluble ICAM-1 binding assay

Isolated murine neutrophils were resuspended in HBSS Buffer and stimulated with 100ng/ml rmCXCL1 (PeproTech), PMA (Calbiochem) or an equal amount of HBSS buffer in the presence of soluble rmICAM-1 (ICAM-1 hFC chimera; R&D Systems, 20µg/ml), goat anti-human Fcg-biotin (eBioscience) and streptavidin-PerCP-Cy5.5 (eBioscience) for 3 minutes at 37°C. Cells were fixed (FACS Lysing Solution; BD), stained with rat anti-mouse Ly6G-Pacific Blue antibody (1A8; BioLegend) and measured using a flow cytometer.
